# CD11c^+^ myeloid cell exosomes reduce intestinal inflammation during colitis

**DOI:** 10.1172/jci.insight.159469

**Published:** 2022-10-10

**Authors:** Kaylyn M. Bauer, Morgan C. Nelson, William W. Tang, Tyson R. Chiaro, D. Garrett Brown, Arevik Ghazaryan, Soh-Hyun Lee, Allison M. Weis, Jennifer H. Hill, Kendra A. Klag, Van B. Tran, Jacob W. Thompson, Andrew G. Ramstead, Josh K. Monts, James E. Marvin, Margaret Alexander, Warren P. Voth, W. Zac Stephens, Diane M. Ward, Aaron C. Petrey, June L. Round, Ryan M. O’Connell

**Affiliations:** 1 Department of Pathology, Division of Microbiology and Immunology, University of Utah, Salt Lake City, Utah, USA.; 2 University of Utah Flow Cytometry Core, Salt Lake City, Utah, USA.; 3 Department of Internal Medicine, Division of Gastroenterology, and; 4 Hunstman Cancer Institute, University of Utah, Salt Lake City, Utah, USA.

**Keywords:** Cell Biology, Immunology, Inflammatory bowel disease, Noncoding RNAs

## Abstract

Intercellular communication is critical for homeostasis in mammalian systems, including the gastrointestinal (GI) tract. Exosomes are nanoscale lipid extracellular vesicles that mediate communication between many cell types. Notably, the roles of immune cell exosomes in regulating GI homeostasis and inflammation are largely uncharacterized. By generating mouse strains deficient in cell-specific exosome production, we demonstrate deletion of the small GTPase *Rab27A* in CD11c^+^ cells exacerbated murine colitis, which was reversible through administration of DC-derived exosomes. Profiling RNAs within colon exosomes revealed a distinct subset of miRNAs carried by colon- and DC-derived exosomes. Among antiinflammatory exosomal miRNAs, miR-146a was transferred from gut immune cells to myeloid and T cells through a Rab27-dependent mechanism, targeting *Traf6*, *IRAK-1*, and *NLRP3* in macrophages. Further, we have identified a potentially novel mode of exosome-mediated DC and macrophage crosstalk that is capable of skewing gut macrophages toward an antiinflammatory phenotype. Assessing clinical samples, *RAB27A*, select miRNAs, and RNA-binding proteins that load exosomal miRNAs were dysregulated in ulcerative colitis patient samples, consistent with our preclinical mouse model findings. Together, our work reveals an exosome-mediated regulatory mechanism underlying gut inflammation and paves the way for potential use of miRNA-containing exosomes as a novel therapeutic for inflammatory bowel disease.

## Graphical abstract

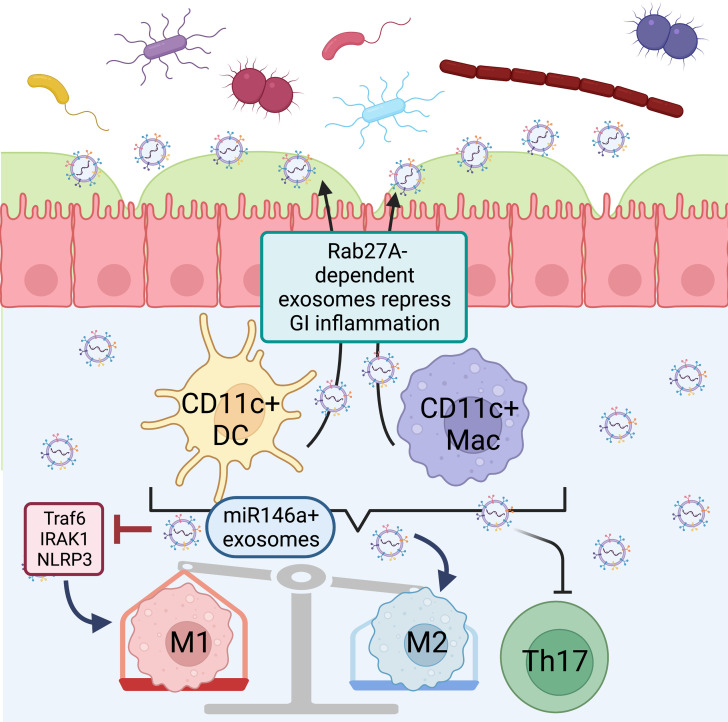

## Introduction

The gastrointestinal (GI) tract harbors a complex immune system that is in constant communication with many cell types and organ systems and that underlies many facets of human health. Maintenance of the GI tract is specifically dependent on proper signaling between the GI immune system and intestinal epithelial cells (IECs). Communication between these cell types is necessary for immune tolerance to commensal microbes, intestinal barrier maintenance, and appropriate responses to pathogenic organisms (1, 2). While our understanding of the immunological mechanisms dictating intestinal homeostasis and the progression of inflammatory diseases in the gut has been under intense investigation in recent decades, many questions regarding the roles of host intercellular signaling remain.

Host extracellular vesicles (EVs) are an important mode of intercellular communication with immunoregulatory properties, yet their functions within the GI tract during health and disease remain largely unclear. IECs, mesenchymal stem cells, and hematopoietic cells all secrete EVs, and populations of these heterogeneous vesicles are readily found in bodily fluids (3, 4). Importantly, distinct EV populations with more immunomodulatory potential were detected in the luminal aspirates of both healthy pediatric and adult controls compared to inflammatory bowel disease (IBD) patients, highlighting their potential importance to disease (5, 6). Although immune populations and their functions change along the length of the human and mouse GI tracts, it is unknown whether EVs from distinct regions or from distinct immune cells or other sources possess different roles in response to intestinal barrier challenges (7, 8). It has also been known that specialized IEC-derived vesicles, termed “tolerosomes,” display MHCII with bound antigen and can travel to lymph nodes and induce tolerance to an orally fed antigen (9–11). Further, exosomes released from apical versus basolateral surfaces of murine IECs differ in their protein composition, suggesting a specialized function for EVs based on the cell of origin or release mechanism (3). However, the identity, contents, and functions of EVs produced by distinct immune cell types within the GI tract remain undefined.

Exosomes, a subset of EVs, are 50 to 200 nm lipid vesicles derived from the multi-vesicular body (MVB) pathway and contain myriad bioactive molecules, including miRNAs (12). miRNAs are small noncoding RNAs that regulate target messenger RNAs by binding their 3′ UTRs to repress translation or reduce stability (13, 14). miRNAs are known to be critical for proper intestinal homeostasis (15, 16). Vesicular trafficking allows for protected intercellular miRNA transfer to regulate target genes in recipient cells, ranging from neighboring cells to those in distal organs (17, 18). The highly stable nature of these vesicular miRNAs makes them appealing as a cell-free therapeutic (19); however, the in vivo functional roles of vesicular miRNAs within the GI tract remains largely uncharacterized.

A particular subset of exosomes is released through a mechanism involving RAB27A and RAB27B, 2 widely expressed small GTPases in the secretory pathway (20–23). RAB27A and RAB27B are functionally redundant in some cell types but are also distinct in other contexts (24–26). With regard to exosome secretion, it is thought that RAB27A is responsible for docking the exosome-containing MVB at the plasma membrane, while RAB27B aids in transferring the MVB from microtubules (23). The dual loss of murine RAB27A and RAB27B results in a chronic inflammatory state and decreased responsiveness to LPS challenge (27), along with defective Treg function (28). Loss of human RAB27A function results in Griscelli syndrome (GS) type 2, wherein patients have a defective immune system and increased risk of viral infection, often requiring BM transplantation in childhood (29). Altered immune cell expression of RAB27A and RAB27B was reported for a cohort of ulcerative colitis (UC) colon biopsy samples, although their functions in this setting was unclear (30). Although it is established that RAB27A/B contribute to exosome release and immune modulation, the individual roles of these GTPases in distinct exosome-producing cell types in vivo as well as their specific functions in the GI tract have been generally unexplored.

Here, we report that whole-body Rab27a and Rab27b double-KO (Rab27DKO) mice have reduced exosome release from colonic tissue. However, we also found that the cellular expression patterns of these 2 genes were largely nonoverlapping. Consequently, we created conditional KO mice for either RAB27A or RAB27B in hematopoietic cells and IECs to examine their specific contributions to GI immune regulation. Using 2 distinct preclinical models of UC, we found increased disease severity in mice lacking RAB27A specifically in hematopoietic or CD11c^+^ myeloid cells. We then found that DC-derived EVs rescued exacerbated colitis in hematopoietic Rab27a-deficient mice. Small RNAs in colon explant– and DC-derived exosomes were profiled to identify exosomal miRNAs potentially responsible for reducing colitis in our mouse model. We found that *miR-146a*, along with a subset of other antiinflammatory miRNAs, were enriched in DC and colonic exosomes and that exosome-mediated *miR-146a* transfer between immune cells within the gut was largely RAB27A dependent. Further, we identified an exosome-dependent mechanism of GI macrophage skewing mediated through uptake of DC-derived exosomal *miR-146a*, a known antiinflammatory miRNA that targets TNF receptor associated factor 6 (TRAF6), interleukin 1 receptor associated kinase 1 (IRAK-1), and NLR family pyrin domain containing 3 (NLRP3), key proinflammatory regulators. Finally, we extended our studies to clinical samples, where we observed dysregulated expression of RAB27A, RNA-binding proteins that can load miRNAs in exosomes, and miRNAs in colon tissues from patients with UC but not Crohn’s disease (CD).

## Results

### RAB27DKO mice display reduced colonic exosome secretion.

**Figure 1:**
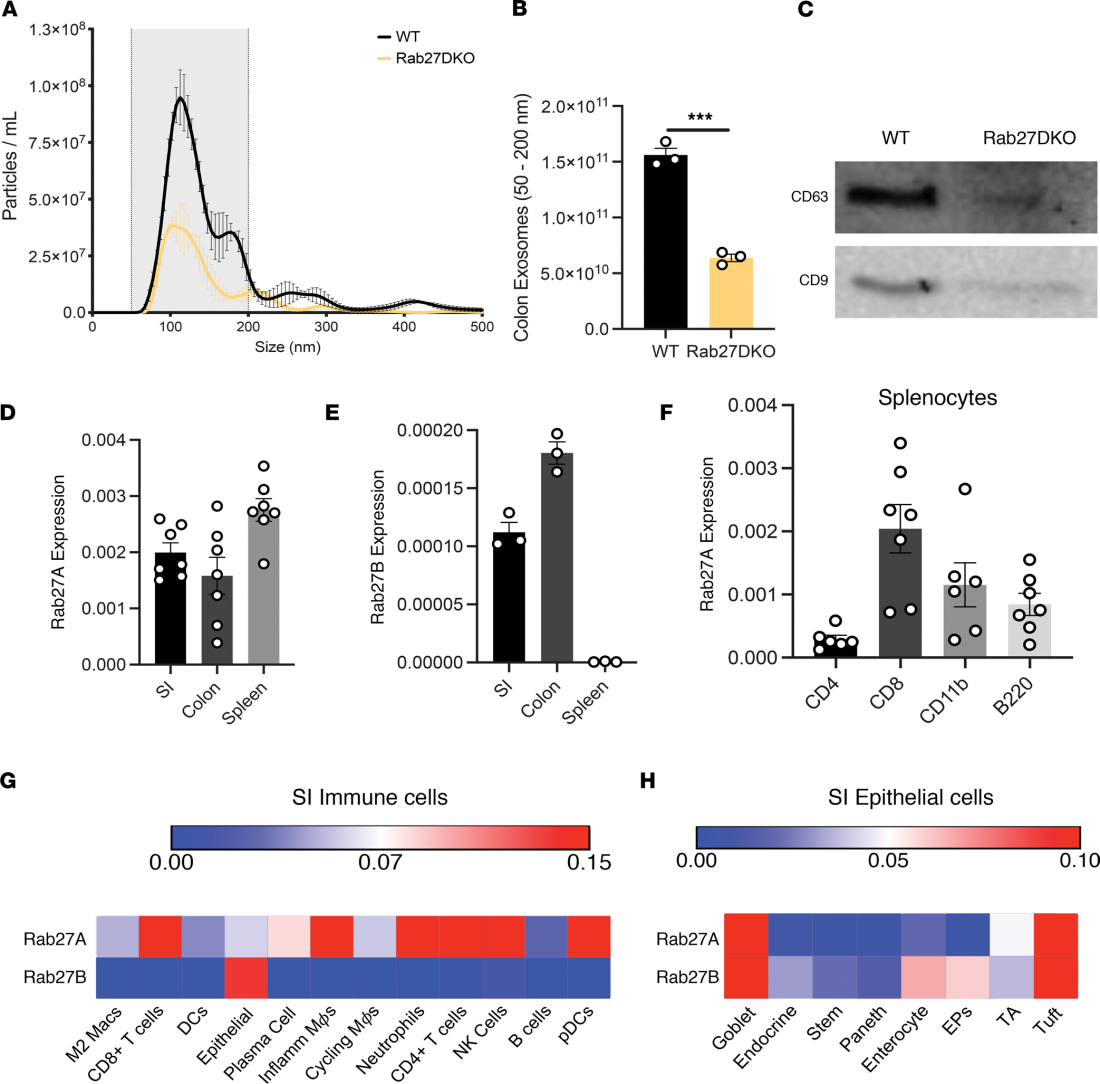
*Rab27A* and *Rab27B* have distinct tissue expression and regulate gut exosome release. (**A**) NTA of WT and Rab27DKO colon explant EVs (*n =* 3). (**B**) Quantification of 50–200 nm particles (the gray shaded area) between genotypes of **A**. (**C**) CD63 and CD9 immunoblot of WT and Rab27DKO colon EVs. (**D**) *Rab27A* (*n =* 7) and (**E**) *Rab27B* expression in distal SI, distal colon tissue, and splenocytes at baseline (*n =* 3). (**F**) *Rab27A* expression in FACS WT CD4^+^, CD8^+^, B220^+^, and CD11b^+^ populations (*n =* 7). (**G**) *Rab27A* and *Rab27B* expression in mouse CD45^+^ SI cells from ref. 31. (**H**) Analysis of mouse CD45^–^ SI cells from ref. 31. Clusters as previously annotated for **G** and **H**. Unpaired 2-tailed *t* test for all bar graphs. ***P < 0.0005. EPs, enterocyte progenitors; TA, transit amplifying cells.

Little is known regarding how exosomes influence intestinal health; therefore, we profiled immune parameters within steady-state colons of Rab27DKO and WT animals. To verify decreased exosome release within the gut, as seen in other cellular compartments of these animals, vesicles were isolated from colonic explants of WT and Rab27DKO mice by differential centrifugation, and their size profiles and concentration were determined using nanoparticle tracking analysis (NTA) (Figure 1A). After quantifying particles in the defined exosome range (50–200 nm) (Figure 1B), and examining known exosome tetraspanin markers (CD63 and CD9) by Western blotting (Figure 1C), we found decreased exosome secretion from the colon of Rab27DKO mice versus controls.

Next, we measured the expression of both GTPases in murine small intestine (SI), colon, and spleen tissues by quantitative PCR (qPCR) to define their expression profiles. Both *Rab27a* and *Rab27b* were highly expressed in the SI and colon, whereas only *Rab27a* was clearly detected in the spleen (Figure 1, D and E), consistent with previous reports of RAB27A and RAB27B protein expression (20). Further, we found that *Rab27a* was expressed in CD4^+^, CD8^+^, B220^+^, and CD11b^+^ populations sorted from the spleen, while *Rab27b* was not detected (Figure 1F, unpublished observations). Mining available murine SI single-cell RNA-sequencing (scRNA-Seq) data sets, we found that *Rab27a* has a diverse expression profile in the hematopoietic compartment of the SI, with detectable expression across many distinct cellular lineages (Figure 1G) (31). *Rab27b* has a more restricted expression profile, with the highest expression limited to the epithelial cluster (Figure 1G). Since both GTPases are expressed in epithelial cells and highly expressed in whole SI and colon tissues, we assessed scRNA-Seq of the murine SI epithelial cell compartment (32). Again, there was a distinct epithelial cell profile, with both *Rab27a* and *Rab27b* being most highly expressed in the tuft, goblet, and enteroendocrine cell clusters versus the other clusters (Figure 1H). This distinct expression of *Rab27a* and *Rab27b* across the intestinal landscape led us to create cell type–specific conditional KO mice to further investigate the individual roles of these 2 GTPases in gut exosome secretion and in regulating intestinal homeostasis and inflammatory responses.

### Rab27a in the hematopoietic compartment modulates inflammatory responses in the GI tract.

**Figure 2:**
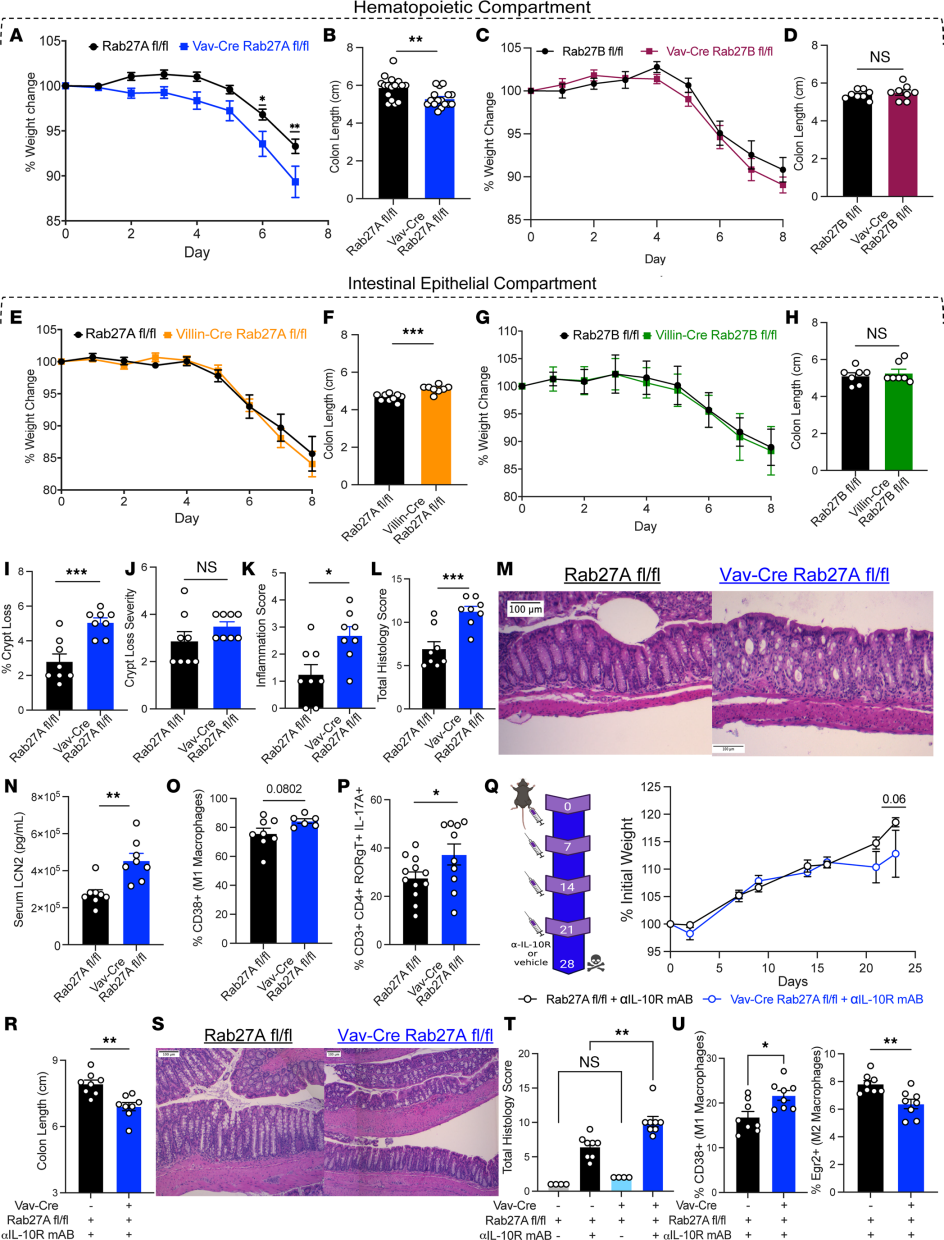
Specific role for hematopoietic Rab27A in the regulation of colitis phenotypes. (**A**) Weight loss of Rab27A^fl/fl^ animals versus Vav-iCre Rab27A^+^ animals during a 7-day course of 2.5% *w/v* DSS (*n =* 18). (**B**) Colon length of animals from **A**, with data representative of 3 combined independent experiments. (**C**) Weight loss of floxed versus Vav-iCre Rab27B^+^ animals during an 8-day DSS course (*n =* 8). (**D**) Colon length of **C**, data representative of 2 independent experiments. (**E**) Weight loss of floxed versus Villin-Cre Rab27A^+^ animals during an 8-day DSS course (*n =* 9). (**F**) Colon length of **E**. (**G**) Weight loss of floxed versus Villin-Cre Rab27B^+^ animals during an 8-day DSS course (*n =* 7). (**H**) Colon length of **G**. (**I**) Percentage of colon crypt loss between genotypes of **A**. (**J**) Severity of colonic crypt damage of **A**. (**K**) Inflammation score of DSS-treated colons of **A**. (**L**) Total colon histology score of **I**–**K** (*n =* 8). (**M**) Representative H&E-stained colons from **A**. (**N**) Serum LCN2 levels of mice from **A** (*n =* 8). (**O**) Percentage of CD38^+^ “M1-like” macrophages in cLP of floxed and Vav-Rab27A cKO mice from **A**. (**P**) Percentage of cLP Th17 cells from floxed and Vav-Rab27A cKO mice, from **A**. (**Q**) Schematic of αIL-10R mAb experiment and weight loss of floxed or Vav-Cre Rab27A^+^ mice given mAb. (**R**) Colon length of **Q**. (**S**) Representative H&E staining of colons from **R**. (**T**) Total histology scores from **R**. (**U**) Percentage of CD38^+^ M1-like and Egr2^+^ macrophages in cLP of floxed and Vav-Rab27A cKO mice from **Q**. Two-way ANOVA mixed model analysis with Geisser-Greenhouse correction for weight loss graphs. Unpaired 2-tailed *t* test for bar graphs, except **T**. For **T**, 2-way ANOVA with Tukey’s multiple-comparison test comparing the column means. *P < 0.05, **P < 0.005, ***P < 0.0005. Scale bars in all images are 100 μm. See also Supplemental Figures 1 and 2.

Utilizing CRISPR-based approaches, we generated floxed mouse strains targeting *Rab27a* and *Rab27b*. For *Rab27a*, we targeted exon 4 (transcript variant 1), the location of some of the natural point mutations in GS type 2, and confirmed by Southern blot that founder floxed-only mice had correct insertions of *loxP* sites (29, 33) (Supplemental Figure 1, A and B; supplemental material available online with this article; https://doi.org/10.1172/jci.insight.159469DS1). For *Rab27b*, we targeted exons 3 to 4 (transcript variant 2), predicted to result in a nonfunctional protein after deletion, and again confirmed *loxP* insertions by Southern blot (Supplemental Figure 1, C and D). We generated broad hematopoietic cell KO animals for either *Rab27a* or *Rab27b* by crossing these floxed mice with Vav-iCre mice. Next, we challenged both strains with dextran sulfate sodium (DSS) to induce acute colitis. Results indicate that Vav-iCre Rab27a^fl/fl^ animals (Vav-Rab27a cKO) were more susceptible to DSS-induced weight loss and colon shortening, indicating worsened disease than floxed littermate controls. This suggests a role for hematopoietic cell–derived exosomes in maintaining inflammation (Figure 2, A and B). The use of littermate controls aided us in controlling for microbiota differences between the groups for this experiment and subsequent experiments by exposing all pups to the same initial microbiota. In contrast to the Vav-Rab27a cKO, Vav-iCre Rab27b^fl/fl^ (Vav-Rab27b cKO) mice did not show a change in weight or colon length compared to littermate controls during colitis experiments (Figure 2, C and D).

Due to the detection of *Rab27a* and *Rab27b* in IECs by scRNA-Seq and qPCR, we investigated the contribution of epithelial cell–derived *Rab27a* and *Rab27b* to the maintenance of the intestinal barrier and response to inflammatory challenge. Floxed animals were crossed with Villin1-Cre mice and subjected to DSS-induced colitis. We found that knocking out either *Rab27a* or *Rab27b* in the IECs did not affect DSS-induced colitis weight loss (Figure 2, E–H). Villin-Cre Rab27a^fl/fl^ animals had modestly longer colons after challenge, potentially indicating some resistance to colitis challenge (Figure 2F). Villin-Cre Rab27b^fl/fl^ animals displayed no change in colon length, indicating that *Rab27b* within the IEC compartment is not modulating this particular response (Figure 2H). Collectively, these results indicate a critical role for hematopoietic derived *Rab27a* in limiting disease during DSS-induced colitis.

Next, we further characterized the colons of Vav-Rab27A cKO mice after DSS colitis. By H&E staining, we observed a higher incidence of crypt loss, worsened crypt destruction, and increased inflammation versus WT controls. This resulted in an elevated total histology score, indicating increased disease severity (Figure 2, I–L). Representative images of this pathology are shown (Figure 2M). Additionally, we measured serum lipocalin-2 (LCN2) levels, which correspond with disease state, and found higher LCN2 in Vav-Rab27A cKO animals versus WT controls (Figure 2N). To determine the relevant immune cell type(s) responsible for exacerbating colitis within Vav-Rab27a cKO animals, we examined the immune cells present during colitis in Vav-Rab27A cKO mice compared with floxed controls. There was an increased proportion of CD38^+^ “M1-like” macrophages and elevated Th17 cells in the colonic lamina propria (cLP) of Vav-Rab27A cKO mice after DSS colitis (Figure 2, O and P). Proper *Rab27a* deletion in Vav-Rab27a cKOs was validated by qPCR, by Western blot, and by a subsequent decrease in hematopoietic exosome secretion measured by NTA (Supplemental Figure 2, A–C).

Because a colitis phenotype associated with Villin-Cre Rab27a/b deletion was not observed, we wanted to confirm that all other Rab27 cKOs were functioning as expected. Using Western blots and qPCR, we confirmed deletion of RAB27A or RAB27B with Villin1-Cre (Supplemental Figure 2, D–G). Additionally, *Rab27b* was not upregulated upon loss of *Rab27a* in immune cells, suggesting no functional compensation between the conditions analyzed (unpublished observations). Further, other Rab GTPases (*Rab2b*, *Rab5a*, and *Rab9a*) were not upregulated despite being potentially involved in exosome secretion in HeLa cells (23). This highlights Rab27a’s importance to this process of suppressing inflammation in the gut during colitis. We also did not observe any obvious changes to baseline phenotypes in the guts of any of these mice as indicated by no changes to the colon lengths of these Rab27 cKOs versus floxed controls (Supplemental Figure 2H) as well as no significant alterations to pertinent immune cell populations in the thymus, mesenteric lymph node (mLN), spleen, or cLP at baseline (unpublished observations). Importantly, there were also no changes to macrophage populations, as defined by CD38 and early growth response 2 (Egr2) markers, at steady-state between floxed and cKO animals (Supplemental Figure 2I).

Having observed a critical role for hematopoietic RAB27A during DSS-induced colitis, we wanted to confirm that this phenomenon occurred in a second model of colitis. To do this, we utilized the anti–IL-10 receptor (αIL-10R) blocking mAb model of IBD (34). This model is akin to the IL-10^–/–^ model of spontaneous colitis but occurs more frequently and at a quicker pace than the KO animal model (35). Four weekly i.p. injections of αIL-10R mAbs were administered (Figure 2Q). We found that Vav-Rab27A cKO mice had increased weight loss and shorter colon lengths compared with floxed controls that also received the mAb (Figure 2, Q and R). No differences between the 2 groups were observed upon vehicle administration alone (Supplemental Figure 2J). Additionally, histological analysis indicated that Vav-Rab27A cKO had a greater occurrence of epithelial hyperplasia, goblet cell loss, and inflammatory aggregates as reflected in the total histology score, which is common for this model of colitis (Figure 2, S and T) (36, 37). Representative images of the mAb colons and quantification of the inflammatory aggregates are shown (Supplemental Figure 2K). Finally, we observed a similar shift in macrophage populations in Vav-Rab27A cKO mouse colons similar to DSS colitis, with more “M1-like” macrophages compared with “M2-like” macrophages (Figure 2U). Cumulatively, these results indicate that hematopoietic RAB27A function is crucial for regulating the inflammatory response during IBD.

### Rab27a functions in myeloid cells to release exosomes that suppress DSS-induced colitis.

**Figure 3:**
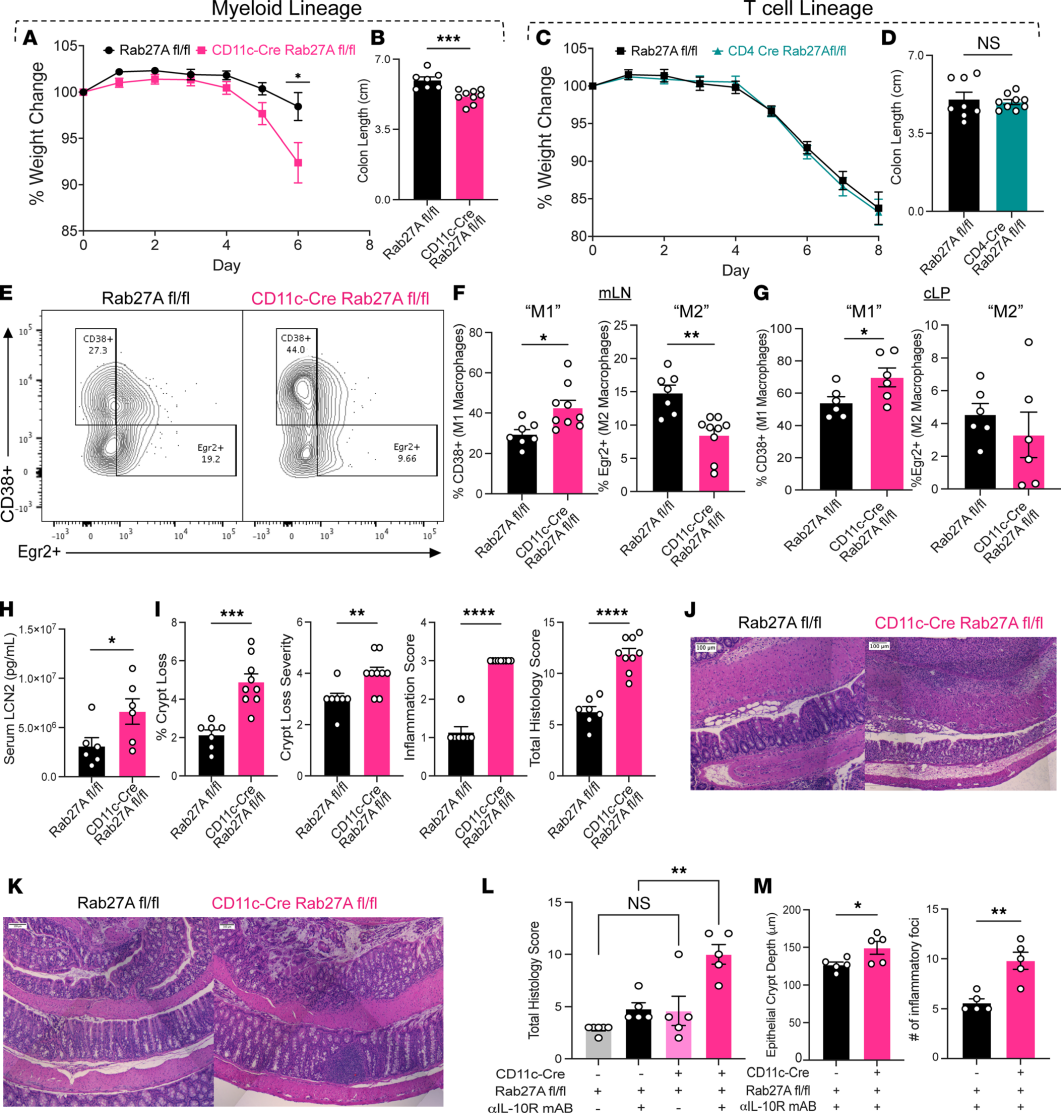
Rab27A functions in CD11c^+^ myeloid cells to regulate colitis phenotypes. (**A**) Weight loss of floxed animals versus CD11c-Cre Rab27A^+^ animals during a 6-day DSS course. (**B**) Colon length of **A** (*n =* 9), data representative of 4 independent experiments. (**C**) Weight loss of floxed versus CD4-Cre Rab27A^+^ animals during an 8-day DSS course (*n =* 9). (**D**) Colon length of **C**. (**E**) Representative flow plot of macrophage gating of **F** and **G**. (**F**) Percentage of CD38^+^ M1-like macrophages and of Erg2^+^ M2-like macrophages in floxed and CD11c-Rab27A cKO mLNs after DSS (*n =* 9). (**G**) Percentage of CD38^+^ M1-like macrophages and of Egr2^+^ M2-like macrophages in WT and CD11c-Rab27A cKO cLP after DSS (*n =* 6). (**H**) Serum LCN2 levels of mice from **A** as measured by ELISA (*n =* 6). (**I**) Percentage of colon crypt loss, severity of colon crypt loss, inflammation score, and total histology score of H&E-stained colons of **A** (*n =* 9). (**J**) Representative H&E-stained colons from **A**. (**K**) Representative H&E staining from WT and CD11c-Rab27A cKO mAb mice. (**L**) Total histology scores of **K**. (**M**) Quantification of epithelial crypt depth and number of inflammatory aggregates in **K**. Two-way ANOVA mixed model analysis with Geisser-Greenhouse correction for weight loss graphs. Unpaired 2-tailed *t* test for bar graphs except **L**. For **L**, 2-way ANOVA with Tukey’s multiple-comparison test comparing the column means. *P < 0.05, **P < 0.005, ***P < 0.0005. Scale bars in all images are 100 μm. See also Supplemental Figure 3.

Previous in vitro literature indicates an immune-regulatory role for DC- and T cell–derived EVs, and our observed alterations of the myeloid and T cell compartments in Vav-Rab27a cKO mice during intestinal inflammation prompted us to generate more specific cKO animals to identify relevant immune cell types involved in worsened colitis in the absence of hematopoietic Rab27a. Rab27a^fl/fl^ mice were crossed with CD11c-Cre and CD4-Cre mouse strains to examine the relative contributions of Rab27a within the innate and adaptive arms of immunity. Decreased *Rab27a*/RAB27A expression were confirmed via qPCR and Western blot in both cKO animals (Supplemental Figure 3, A and B). While CD4-Cre Rab27a^fl/fl^ mice displayed no difference compared to controls during DSS colitis or at baseline (Supplemental Figure 3C), CD11c-Cre Rab27a^fl/fl^ animals (CD11c-Rab27A cKO) exhibited greater weight loss and colonic shortening following DSS treatment, indicating that myeloid cells, including DCs and some macrophages, within the hematopoietic compartment utilize RAB27A to restrict DSS colitis phenotypes (Figure 3, A–D).

Profiling of CD11c-Rab27a cKO animals demonstrated altered macrophage populations that included increases of CD11c^–^ “M1-like” proinflammatory CD38^+^ macrophages and decreased CD11c^–^Egr2^+^ “M2-like” antiinflammatory macrophages, compared with floxed littermate controls in the mLNs and cLP (Figure 3, E–G). Elevated serum LCN2 levels and increased epithelial damage, as shown in representative images, were observed in CD11c-Rab27a cKO mice versus controls, consistent with worsened disease (Figure 3, H–J). No significant alterations to the macrophage populations within the homeostatic colons of CD11c-Rab27a cKO were observed, nor were any alterations to colon length, spleen weight, or fecal LCN2 levels (Supplemental Figure 3, D–F). We also assessed the response of these CD11c-Rab27a cKO and floxed mice to the αIL-10R mAb model of IBD and saw a phenocopy of the histological changes seen in Vav-Rab27A cKO mice challenged with this same model (Figure 3, K and L), including increased epithelial crypt depth and total number of inflammatory aggregates in Vav-Rab27A cKO mice (Figure 3M). These data suggest a critical role for Rab27a-dependent, DC-derived exosomes within the intestinal microenvironment during experimental IBD, leading us to examine the molecular cargo carried and delivered by these EVs that could be responsible for this antiinflammatory function of RAB27A within the GI tract.

### DC-derived exosomes containing miRNAs are released in the intestinal microenvironment.

**Figure 4:**
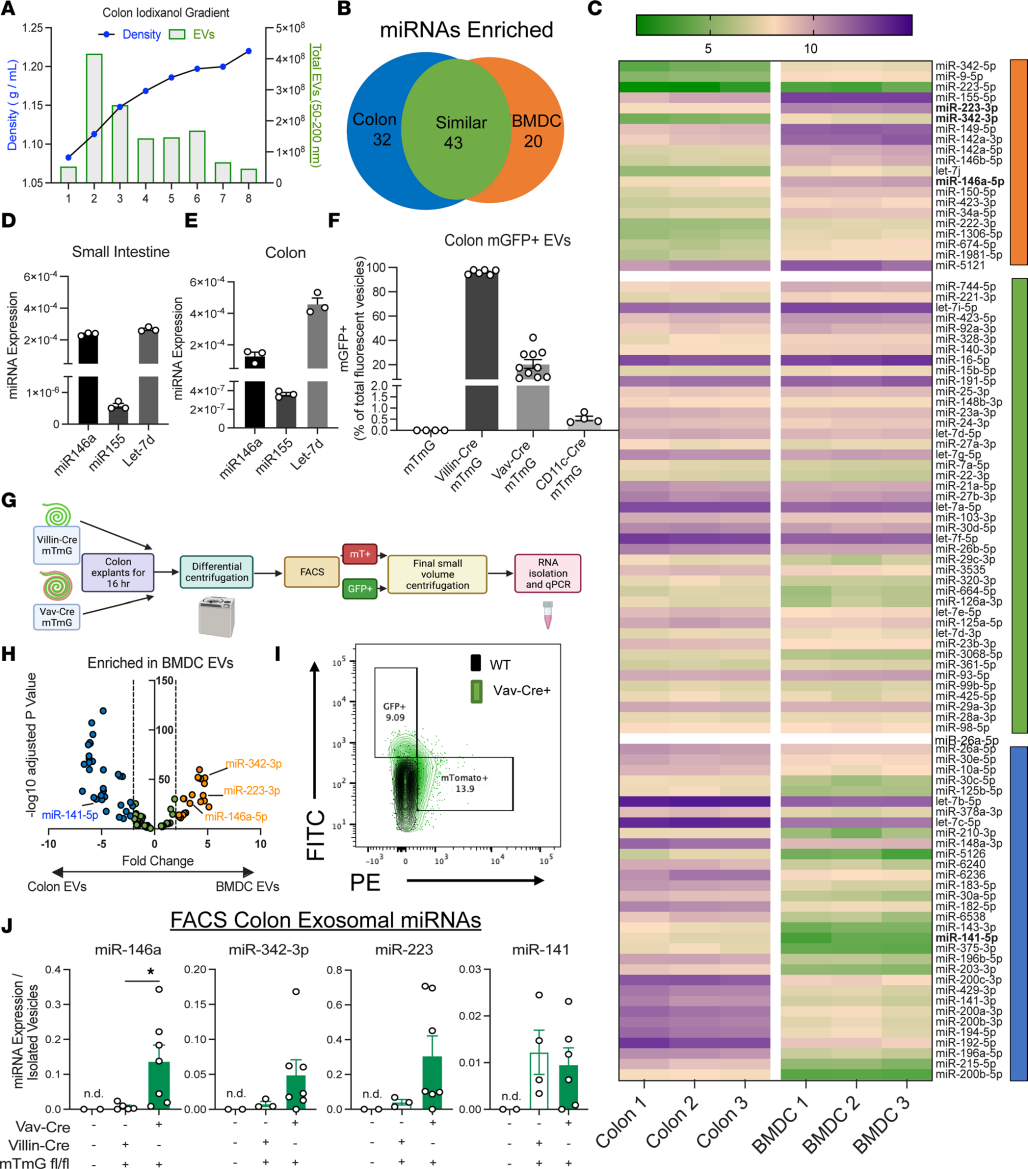
Profiling of miRNAs within colon and BMDC-derived exosomes. (**A**) Density measurements of iodixanol gradient overlaid on top of NTA of each fraction of colon iodixanol gradient. (**B**) Venn diagram of differentially enriched miRNAs between colon EVs and BMDC EVs (*n =* 3). (**C**) Heatmap of the 3 groups within **B**. (**D**) miRNA qPCR confirmation of miR-146a, miR-155, and let-7-d from SI or (**E**) colon EV preps (*n =* 3). (**F**) Percentage of mGFP^+^ vesicles of fluorescent vesicles from mTmG, Villin-Cre mTmG, Vav-iCre mTmG, and CD11c-Cre mTmG mice as measured by Aria II (*n =* 6–10). (**G**) Schematic of flow-sorting vesicles from colon explants of **H** and **I**. (**H**) Volcano plot of differentially expressed miRNAs highlighting miRNA enrichment in BMDC EVs. Dashed line, fold change of –2/2. (**I**) Representative flow plot of sorted mTmG vesicles with overlay of gated vesicles to be sorted from both a WT mouse and Vav-Cre mTmG mouse. (**J**) miR-146a, miR-342-3p, miR-141, and miR-223 expression in GFP^+^ sorted vesicles from Villin-Cre mTmG and Vav-iCre mTmG colon explants (*n =* 7). Data representative of 2 independent experiments. Unpaired 2-tailed *t* test for bar graphs. *P < 0.05. n.d., not detected.

Exosomes are composed of many bioactive molecules, including miRNAs, which are one of the most abundant EV noncoding RNA species (38, 39). Because miRNAs are critical regulators of inflammation and colitis, we examined gut-derived EVs to determine which miRNAs they carry that may have regulatory functions during colitis. Because we saw defects in inflammation control when DCs could not release exosomes, we compared colon EV miRNAs to BM-derived DC (BMDC) EV miRNAs to determine which miRNAs were present in both BMDCs and in a highly heterogenous colon explant EV preparation. To do this, exosomes were purified from BMDCs or colon explants with an iodixanol gradient, followed by miRNA sequencing (Figure 4A). Thirty-two miRNAs were enriched in colon EVs compared with BMDC EVs, 20 were enriched in BMDC EVs compared with colon EVs, and 43 were roughly equivalent in exosomes from the 2 sources (Figure 4, B and C; NCBI Gene Expression Omnibus [GEO] accession: GSE190854). We verified the presence of 3 miRNAs from our sequencing, *miR-146a*, *miR-155*, and *Let-7d*, within SI and colon explant exosome preparations by qPCR, highlighting their potential importance in the gut (Figure 4, D and E).

Next, we repurposed a murine model to track the cellular source of colonic exosomal miRNAs. We developed several types of mTmG reporter mice by crossing mTmG mice with Villin1-Cre, Vav-iCre, and CD11c-Cre to produce mouse strains with cell-specific plasma membrane GFP-labeled exosomes. With this system, GFP^+^ vesicles originate from Cre^+^ cells that have recombined the lox-mTomato-stop-lox system upstream from N-terminus membrane tagged GFP within the Rosa locus of these mice (40). The default fluorescence of Cre^–^ cell plasma membranes is mTomato, while Cre^+^ cells label their membranes with GFP, including exosomal membranes. A large percentage of colon explant vesicles were derived from IECs, as expected. A substantial proportion of vesicles were also hematopoietic cell derived, while a small percentage of vesicles were from CD11c^+^ cells (Figure 4F). With this system, we were able to track the cellular sources of the miRNAs found in colon EV preparations. Using a FACSAria II cell sorter (BD), GFP^+^ EVs were sorted from colon explants of either Villin1-Cre mTmG mice or Vav-iCre mTmG mice, and RNA was isolated from these exosomes (Figure 4G). Using qPCR, we assayed 3 antiinflammatory miRNA candidates from our sequencing results that included *miR-146a*, *miR-342-3p*, and *miR-223-3p*, all of which are thought to regulate NF-κB (41–43). Each was demonstrated to be enriched in Vav-iCre mTmG GFP^+^ vesicles, consistent with hematopoietic, and potentially DC, origin (Figure 4, H–J). We also observed unchanged *miR-141* between GFP^+^ vesicle populations from either Villin-Cre or Vav-iCre origin as a control (Figure 4J). Of note, we were unable to recover enough RNA from sorted CD11c^+^ cell–derived exosomes for downstream analysis by qPCR. Collectively, these results indicate that selected exosomal miRNAs originate from a hematopoietic source, which includes CD11c^+^ dendritic and other myeloid cells.

### Rab27a-dependent transfer of exosomal miR-146a to gut macrophages and T cells in vivo.

**Figure 5:**
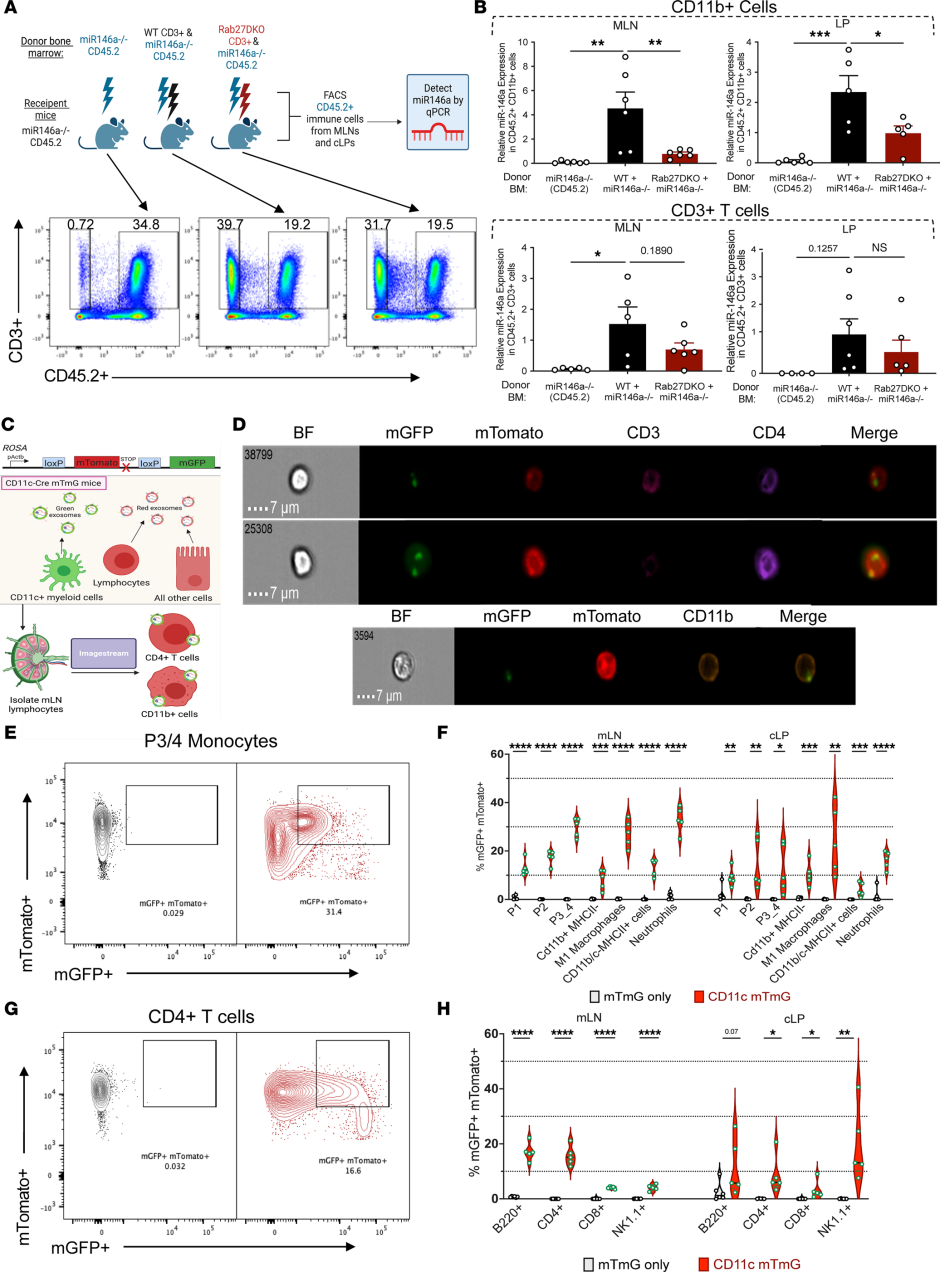
Rab27-dependent transfer of miR-146a to myeloid and T cells in vivo. (**A**) Schematic of BM chimera and representative flow plots of CD45.2^+^CD3^+^ sorted cells from the mLN. (**B**) miR-146a expression within sorted mLN and cLP CD11b^+^ cells, as well as mLN and cLP CD4^+^ T cells (*n =* 6). One-way ANOVA with Tukey’s multiple-comparison test comparing the column means. (**C**) Schematic of mTmG gut explant experiment with ImageStream technology. (**D**) Representative images of green puncta on mTomato^+^CD3^+^CD4^+^ cells from CD11c-Cre mTmG mLNs. Representative images of green puncta on mTomato^+^ CD11b^+^ cells from CD11c-Cre mTmG mLNs. (**E**) Representative flow cytometry plots of P3/4 monocytes from **F**. (**F**) Percentage of mGFP^+^mTomato^+^ myeloid populations within the mLNs and cLP. (**G**) Representative flow cytometry plots of CD4^+^ T cells from **H**. (**H**) Percentage of mGFP^+^mTomato^+^ lymphocyte populations within the mLNs and cLP. Unpaired 2-tailed *t* test for each cell type in **F** and **H**. *P < 0.05, **P < 0.005, ***P < 0.0005. See also Supplemental Figure 4.

After detecting *miR-146a* at high levels in GFP^+^ colon EVs from Vav-iCre mTmG mice, and knowing its importance in the gut and overall immune regulation (41–43), we developed an in vivo system to detect possible Rab27-dependent *miR-146a* transfer and a way to determine recipient cell identity. We utilized BM chimeras, wherein miR-146a^–/–^ CD45.2 irradiated animals were the recipients of miR-146a^–/–^ CD45.2 BM, a 1:1 mix of WT CD45.1 and miR-146a^–/–^ CD45.2 BM, or a 1:1 mix of Rab27DKO CD45.1 and miR-146a^–/–^ CD45.2 BM (Figure 5A and Supplemental Figure 4A). After reconstitution, FACS was used to isolate miR-146a^–/–^ CD45.2^+^ immune cell subsets from mLNs and cLP and qPCR used to detect transferred *miR-146a* after verifying BM reconstitution (Figure 5A). *miR-146a* was detected in miR-146a^–/–^ CD45.2 CD11b^+^ myeloid and CD3^+^ T cells in both the mLNs and cLP, indicating RAB27-dependent transfer of *miR-146a* in vivo (Figure 5B).

With this intercellular transfer of miR-146a determined, we wanted to further demonstrate that GFP^+^ vesicle-like lipid puncta from CD11c-Cre mTmG mice were associating with CD11b^+^ and CD4^+^ cells within the mLNs (Figure 5C and Supplemental Figure 4B). Utilizing ImageStream technology, we observed these GFP^+^ puncta on CD3^–^CD11b^+^ and CD3^+^CD4^+^mTomato^+^ cells within the mLNs, indicating that lipids originating from CD11c^+^ cells were taken up by CD4^+^ T and CD11b^+^ myeloid cells (Figure 5, D and E). Importantly, we did not see any green vesicle-like puncta in CD11c-Cre^–^ mTmG^fl/fl^ control mLN samples (Supplemental Figure 4C). These data support the hypothesis that CD11c cell–derived vesicles, containing miR-146a, are delivered to myeloid and T cell populations within gut lymph nodes and intestinal tissues.

Finally, to better understand the overall gut immune cell repertoire capable of receiving these vesicles, we utilized traditional flow cytometry to detect CD45^+^CD11c^–^mTomato^+^ cells that were also membrane GFP^+^ (mGFP^+^), consistent with receiving GFP lipid membranes from CD11c^+^ DCs and macrophages. We examined both the mLNs and cLP of mTmG and CD11c-Cre^+^ mTmG mice and saw distinct immune patterns based on anatomic location. Within the myeloid lineage in the mLNs, there was a preference for P3/4 monocytes, “M1-like” macrophages, and neutrophils to be mGFP^+^ (Figure 5, E and F). Interestingly, this pattern shifted in the cLP to include more monocyte lineages, and there was less mGFP positivity in the neutrophil populations. Importantly, we also looked at the adaptive immune system and saw that within the mLNs, CD4^+^ and B220^+^ cells were readily capable of being mGFP^+^ whereas in the cLP, this shifted to also include natural killer (NK) cells (Figure 5, G and H). Together, these results indicate that CD11c^+^ DCs and macrophages in the gut are able to transfer GFP-labeled lipids to a variety of immune cells in the GI tract.

### DC-derived exosomal miR-146a is delivered to gut macrophages and alters their skewing in vivo.

**Figure 6:**
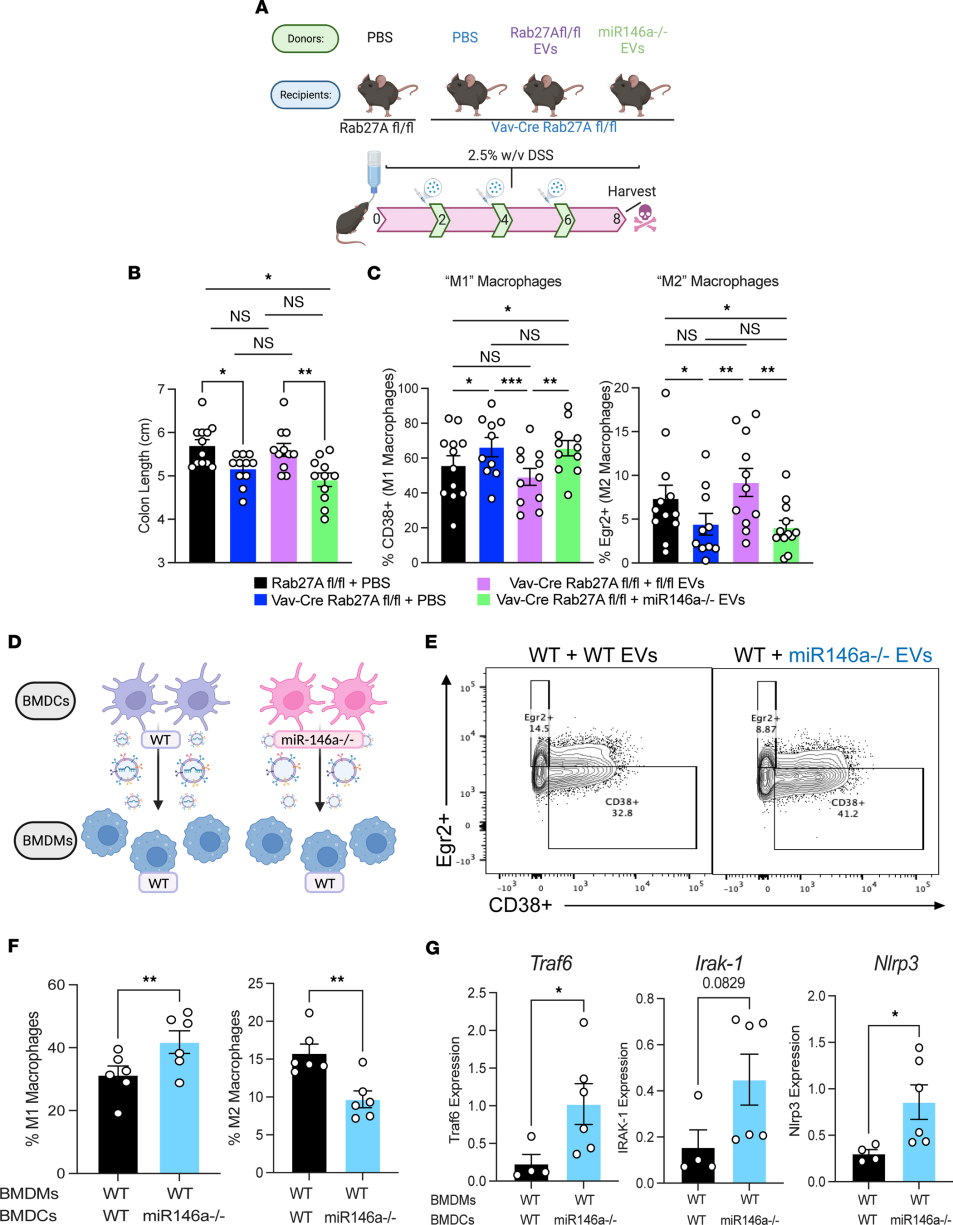
In vivo administration of miR-146a by CD11c^+^ myeloid cell–derived exosomes regulates macrophage skewing during colitis. (**A**) Schematic of exosome rescue experiment. (**B**) Colon length of floxed + PBS, Vav-Rab27A cKO + PBS, Vav-Rab27A cKO + WT EVs, and Vav-Rab27A cKO + miR146a^–/–^ EVs after a 7-day DSS course (*n* = 12). Data are representative of 2 independent experiments. (**C**) Percentage of CD38^+^ M1-like macrophages in groups from **B**. Percentage of Egr2^+^ M2-like macrophages in groups from **B** (*n =* 12). (**D**) Schematic of in vitro transfer of BMDC EVs to BMDMs. (**E**) Representative flow plots of CD38^+^ and Egr2^+^ macrophages quantified in **F**. (**F**) Percentage of CD38^+^ M1-like macrophages and Egr2^+^ M2-like macrophages. (**G**) qPCR analysis of BMDMs given either WT or miR146a^–/–^ BMDC EVs, looking at targets of miR-146a. Unpaired 2-tailed *t* test for bar graphs with 2 groups. Two-way ANOVA with Tukey’s multiple-comparison test comparing the column means for **B** and **C**. *P < 0.05, **P < 0.005, ***P < 0.0005.

RAB27A has been implicated in other secretory functions besides exosome secretion; therefore, we tested whether RAB27A-dependent myeloid cell–derived exosomes were involved in the worsened colitis phenotype. Additionally, the therapeutic potential of exosomal miRNAs holds great promise, as they would be based on a natural delivery system that has been optimized by evolution. To examine the functional impact of exosomal *miR-146a* and determine its efficacy as a potential IBD therapy, we administered vehicle (PBS) or approximately 10^9^ WT or miR-146a^–/–^ BMDC exosomes i.p. on days 2, 4, and 6 to mice challenged with DSS colitis over 8 days (Figure 6A). We observed shorter colons in Vav-Rab27a cKO animals that received miR-146a^–/–^ exosomes than in those that received WT, miR-146a–sufficient exosomes (Figure 6B). These shorter colons were akin to KO mice given vehicle, whereas the WT exosomes restored KO colon length to near WT length. At the cellular level, CD38^+^ M1-like proinflammatory macrophages were at higher levels (Figure 6C), and EGR2^+^ M2-like antiinflammatory macrophages were at lower levels in mice that received miR-146a^–/–^ versus WT exosomes (Figure 6C). These results phenocopy the macrophage profile observed in Rab27a cKO mice and show that the administration of WT exosomes to Rab27a cKO mice skews these macrophages back to WT proportions of M1- and M2-like populations.

We next sought to identify the coding mRNA direct targets that were repressed by miR-146a in this setting and determine if this macrophage skewing could be replicated in vitro. We generated WT BM-derived macrophages (BMDMs) and BMDCs from WT and miR-146a^–/–^ mice over the course of 7 days. After this differentiation period, we isolated exosomes from the DCs (either WT or miR-146a^–/–^) and administered them to WT macrophages without any other stimulus to determine if *miR-146a* was sufficient to skew the BMDMs as we had seen in vivo (Figure 6D). Results indicated that these BMDMs were skewed by the presence or lack of *miR-146a* in the exosomes. When *miR-146a* was lacking from the exosomes, BMDMs were more M1-like than when the miRNA was present (Figure 6, E and F). Interestingly, we were also able to see that without *miR-146a* present in exosomes, WT BMDMs had increased levels of *Traf6*, *Irak-1*, and *Nlrp3* transcripts (Figure 6G). These proinflammatory mediators are known targets of *miR-146a* and illustrate the functional capacity of the miRs loaded into these exosomes. Collectively, these data provide evidence that *miR-146a* can be transferred in an RAB27-dependent manner from CD11c^+^ myeloid cells to gut immune cells to regulate macrophage populations known to drive inflammation during DSS colitis.

### Dysregulation of genes critical to exosome release, loading, and increased miRNA expression in patients with IBD.

**Figure 7:**
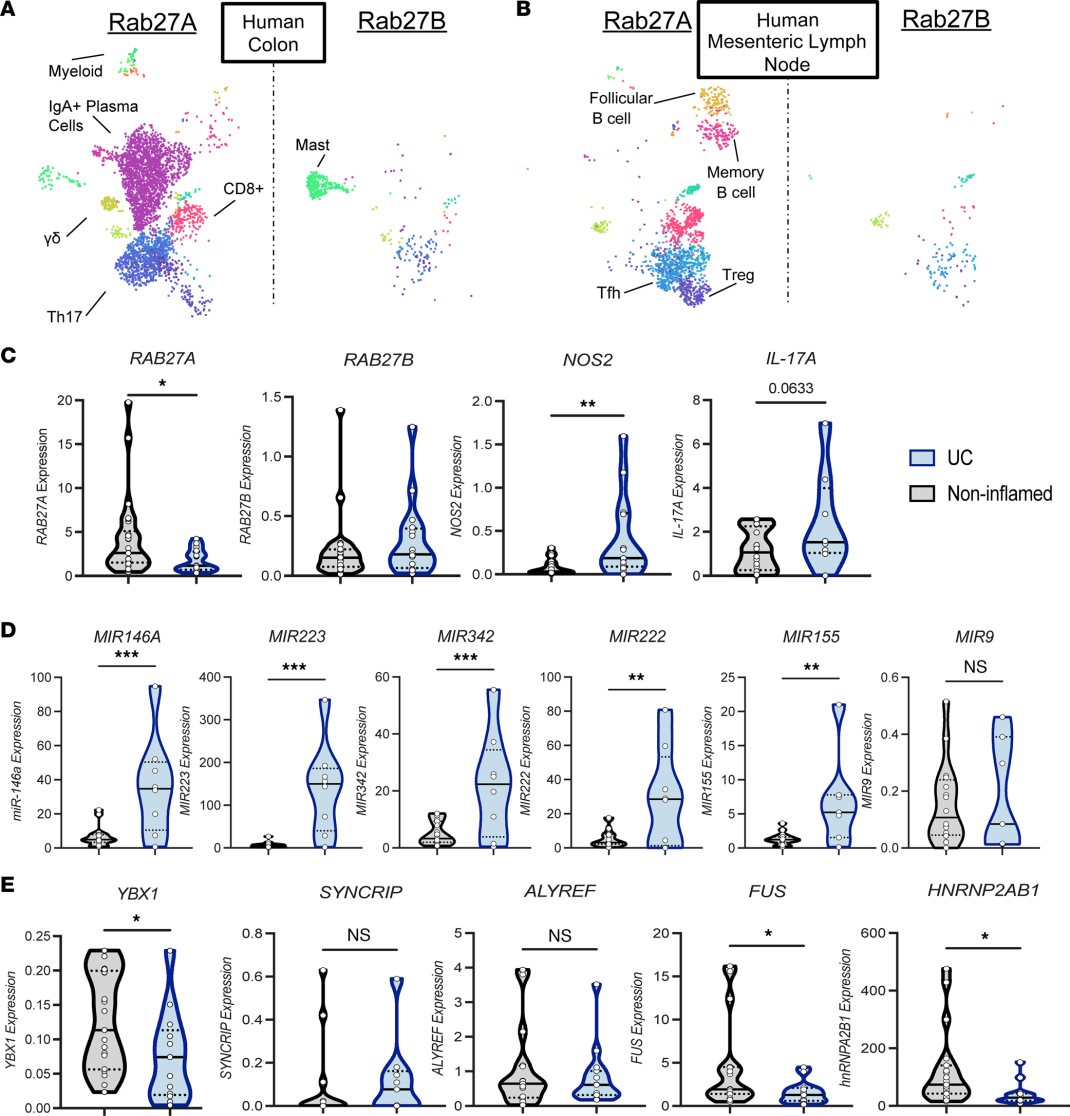
Dysregulation of RAB27A, inflammatory genes, and miRNA signatures in patients with UC. (**A**) Analysis of *RAB27A* and *RAB27B* expression in human baseline colon samples from the Gut Cell Atlas wherein a dot represents detection of transcript (7). Clusters as previously annotated. (**B**) Analysis of *RAB27A* and *RAB27B* expression in human baseline mLN samples from the Gut Cell Atlas (7). (**C**) qPCR analysis of *RAB27A*, *RAB27B*, *IL-17A,* and nitric oxide synthase 2 (*NOS2*) in non-IBD (*n =* 27) and UC patient (*n =* 17) colonic samples. (**D**) miRNA qPCR analysis of miR-146a, miR223, miR-342, miR-222, miR-155, and miR-9 in **C**. (**E**) qPCR analysis of *YBX1,* *HNRNP2AB1*, *FUS, ALYREF,* and *SYNCRIP* in **C**. Quartiles marked with dashed lines. Unpaired 2-tailed *t* test for all violin plots. *P < 0.05, **P < 0.005, ***P < 0.0005. See also Supplemental Figure 5.

We next explored whether these findings were applicable to human patients with IBD. The Gut Cell Atlas scRNA-Seq data set was mined to determine *RAB27A* and *RAB27B* expression from immune cells isolated from nondiseased human colons and mLNs. Similar to mice, expression profiles of *RAB27A* and *RAB27B* were distinct. Within the colon, *RAB27A* was largely detected in IgA^+^ plasma cells, Th1 and Th17 cells, γδ^+^ T cells, myeloid cells, and CD8^+^ T cells (Figure 7A). Within the mLNs, we observed the highest *RAB27A* expression in follicular and memory B cells, NK cells, T follicular helper (Tfh) cells, central memory T cells, CD8^+^ T cells, and Tregs (Figure 7B). This dichotomous expression highlights the diverse roles of this GTPase and the importance of its function within the intestinal compartment. *RAB27B* also displayed a distinct expression pattern, with the highest expression in the mast cell cluster and dispersed expression in colonic T cell clusters (Figure 7A). Within the mLNs, *RAB27B* expression increased in NK cells and shifted to other T cell clusters such as Tfh cells (Figure 7B). Importantly, the cell clusters *RAB27A* and *RAB27B* are detected within are dysregulated in patients with IBD according to scRNA-Seq, suggesting disease relevance of these 2 small GTPases (44).

Next, we assessed a cohort of 17 UC, 10 CD, and 27 noninflamed nondiseased patient samples to determine potential changes in *RAB27A* and *RAB27B* expression during disease (Table 1). Significantly decreased *RAB27A* expression was observed within patients with UC compared with nondiseased patient tissue, while no difference in *RAB27B* expression was found, consistent with results from our mouse model that *RAB27A* is a critical regulator of gut inflammation (Figure 7C). We assessed if this was specific to UC and found that *RAB27A* expression was not altered in patients with CD compared to noninflamed, nondiseased controls. However, *RAB27B* was increased, suggesting context-dependent effects on their expression (Supplemental Figure 5A). We speculate that this reduction in *RAB27A* expression affects exosome-mediated modulation of the immune response to UC. Consistent with this, increased *IL-17A* and *NOS2* expression was observed in patients with UC and CD, suggesting an increase in Th17 cells and “M1-like” macrophages, similar to our Vav-Rab27A cKO mice (Figure 7C and Supplemental Figure 5A).

Next, expression of several miRNAs in the different patient samples was assayed. Increased expression of *MIR-146A* and *MIR223* was observed, 2 miRNAs previously shown to be dysregulated in patients with UC (41, 45) (Figure 7D). Further, increased *MIR342*, *MIR222*, and *MIR155* expression was also observed in patients with UC compared with nondiseased patients, similar to the observed increases in these miRNAs in BMDC-derived exosomes (Figure 4J and Figure 7D). Finally, miR-9 was unchanged between groups, highlighting that only a subset of miRNAs found in exosomes are dysregulated in patients with UC. Interestingly, although there was a distinct increase of these miRNAs in patients with UC, with the exception of *MIR223*, no difference was found in patients with CD, suggesting that there could be a distinct mechanism of action for exosomes within this disease that remains to be elucidated (Supplemental Figure 5). Further, one could interpret this increase in miRNA levels to indicate buildup of these miRNAs within cells with reduced Rab27A expression and thus impaired exosome release.

In recent years, it has been appreciated that the sorting of miRNAs into exosomes can be regulated by RNA-binding proteins (RBPs). Due to our observed preferential sorting of *miR-223* into mouse DC-derived exosomes and the alterations to *RAB27A* and miRNA levels, we also determined if the known *miR-223* exosome sorting protein, Y-box binding protein 1 (YBX1), as well as other known RBPs, were altered in patients with IBD (46, 47). Interestingly, *YBX1* expression was significantly decreased in both diseased states, suggesting that decreased loading of *miR-223* and possibly other miRNAs by YBX1 may be occurring (Figure 7E). Additionally, we saw specific decreases in expression of the RBPs, *FUS* and *HNRNBPA2B1*, but no changes to *ALYREF* or *SYNCRIP*, despite all of these proteins having reported roles in miRNA sorting into exosomes (Figure 7E and Supplemental Figure 5) (48, 49). This observation leads us to hypothesize that there may be defects in both exosome secretion and miRNA loading in patients with IBD, potentially a unique mechanism behind the progression of these heterogenous diseases.

## Discussion

Utilizing exosomes in a therapeutic manner represents an attractive autologous approach moving forward. Exosomes can be derived from patients’ blood cells, including DCs that can first be expanded and differentiated ex vivo and subsequently administered autologously to deliver molecular cargo with therapeutic benefits (50, 51). However, identifying and characterizing endogenous roles for exosomes and their cargo, such as miRNAs, remains a key step toward acquiring the foundational knowledge required for careful design of effective technologies. Here, we identified a potentially novel mechanism whereby CD11c^+^ myeloid cell–derived, Rab27a-dependent exosomes regulate intestinal inflammation, and this occurs in part through miR-146a–mediated impacts on inflammatory macrophage cellular identity and function in the gut. Of clinical relevance, administration of DC-derived miRNA-containing exosomes was able to rescue Rab27a-dependent colitis phenotypes in mice, and dysregulated RAB27A and miRNA expression was observed in a cohort of patients with IBD. To our knowledge, this is also the first study demonstrating that DC-derived exosomes functionally influence macrophage skewing in the GI tract, an important innate immune subset thought to drive colitis disease (44).

We clearly observed defective exosome release from DCs lacking Rab27A, and this was accompanied by increased DSS colitis disease severity. Additionally, we saw that in a different model of IBD based on blocking IL-10R, we observed an exacerbated colitis disease phenotype when Rab27a was deleted from CD11c^+^ DCs and macrophages. This demonstrates that this mechanism is broadly applicable and not specific to one preclinical model. To our surprise, yet demonstrating specificity, we also found that Rab27a and Rab27b were dispensable within the IEC compartment during DSS-induced colitis despite high expression in IECs. We acknowledge that these GTPases may play pivotal roles in other homeostatic or disease contexts in the gut not explored here. Further, we have also ruled out a contribution by Rab27a-dependent, T cell–derived exosomes and Rab27b-dependent, hematopoietic derived exosomes in regulating gut inflammatory responses during DSS colitis. In addition to identifying cell-specific functions for Rab27 proteins, future work using our Rab27-floxed mice will enable us and others to further determine and define Rab27-dependent and Rab27-independent EV populations in vivo.

In addition to selectively blunting EV release using Rab27 cKO mice, it is critical to identify and visualize released exosomes in order to further understand their biology. Exosome researchers have studied the biodistribution of EVs following injection in vivo, but few tools exist to track EVs after their release from the cells of origin in vivo (52). The use of FACS-based methods to detect and purify exosome membrane fluorescence triggered by Cre-mediated labeling using mTmG mice is an emerging strategy (53) and builds on previous methods that used lipid-labeling dyes like PKH67 to sort vesicles (54). This innovative sorting method allowed us to separate IEC-derived exosomes from hematopoietic cell–derived exosomes to determine the source of specific exosome cargo, including miRNA species. We were also able to pair this with ImageStream technology to visualize fluorescently labeled lipids associating with specific cell types in vivo, highlighting the importance of this and other emerging technologies in the EV field (55). In the future, we will combine this reporter system with our Rab27 cKO mouse lines to tease apart the roles of Rab27a and Rab27b in distinct vesicle population release in vivo, which can be tracked as vesicles migrate to target cells. This approach can also be applied to other related conditions, such as colon cancer, where the roles of exosomes from tumors and/or hemopoietic cells in modulating immune responses still confound our understanding of such pathologies despite exciting initial reports (56–58). Additionally, future work on distinct miRNA profiling signatures of purified exosomes, such as we have done here, can be integrated with established miRNA target databases, such as HITS-CLIP (59). These types of analyses will provide additional insight into the functional effects of exosomal miRNAs on target cells. Further, several RBPs, including YBX1 (46, 60), showed reduced expression in IBD patient samples. This finding opens the door to interrogate the relevance of these proteins in intestinal disease. Future work will be needed to unravel the roles these proteins play in loading specific miRNA contents into EVs prior to their release in the gut.

Treating inflammatory disorders through immune regulation in the gut remains a challenging clinical endeavor. For instance, not all patients respond to anti-TNF treatments, and there are potent off-target effects of current cytokine-based immunotherapies (61). The biological efficacy of our exosome delivery experiments in mice undergoing experimental colitis provides the groundwork for future DC-based EV miRNA mimic therapies (62). This could be used to alleviate IBD symptoms caused by aberrant macrophage activation and M1 skewing or inflammatory T cell responses, 2 critical immune populations responsible for IBD pathology (63), and will be improved through targeted loading of specific miRNAs to levels with optimal therapeutic impact. Although we identified exosomal *miR-146a* as a critical regulator of GI macrophage skewing, additional exosomal miRNAs beyond *miR-146a* that are involved in Th17 inhibition in BMDC exosomes remains unclear, along with the functional relevance of additional exosome cargo. We did identify miR-222 as a DC exosome-enriched miRNA, and it has been reported that this miRNA along with miR-221 cell-intrinsically regulates Th17 cells within the gut (64). Future research will allow us to discern if this is an exosome-mediated mechanism of immune regulation as well. Also, of note, RAB27A, YBX1, and the exosomal miRNAs identified in our mouse studies were also dysregulated in a cohort of patients with UC, further supporting the translational potential of our work by suggesting that exosome replacement therapy may replenish antiinflammatory EV levels during disease. Taken together, our study points to exosomes and their cargo as promising targets of next-generation therapeutic strategies to treat inflammatory diseases in the GI tract.

## Methods

### Mice.

All mice are on the C57BL/6 background. Rab27DKO (Rab27a^ash/ash^; Rab27b^–/–^) mice were provided by T Tolmachova and MC Seabra (CEDOC, NOVA Medical School, NMS, Universidade NOVA de Lisboa, Lisbon, Portugal) as previously reported (27). Floxed Rab27a (Rab27a^fl/fl^) and Rab27b (Rab27b^fl/fl^) mice were custom generated with the aid of Biocytogen and CRISPR/Cas9-based technology. For Rab27a, Cas9 cut sites were made flanking exon 4. The upstream guide RNA (gRNA) was GGACCCCCTGTACCGTAAAAGC and the downstream gRNA was GGTCCCGACCAGATGTTCCACC. For Rab27b, Cas9 cut sites were made flanking exons 3 to 4. The upstream gRNA was GGTCTTAAGAATTTATCAACTGG and the downstream gRNA was GGCACTCCTACATCCAAGGC. Following the genomic cuts, approximately 1.5 kb homologous regions containing *loxP* sites were injected into zygotes to create founder mice. Southern blots were used to confirm positivity for the 5′ *loxP* site and no random insertion using an internal probe. Mice were verified by junction PCR, using 5′ forward and 3′ reverse for each gene set, and we confirmed *loxP* insertion and correct excision by PCR. We bred these to Cre driver strains, with the female always being hemizygous for Cre^+^ and the male homozygous for Cre^–^. All Cre driver strains were ordered from The Jackson Laboratory (JAX): Villin1-Cre (JAX 004586), CD4-Cre (JAX 022071), CD11c-Cre (JAX 008068), and Vav-iCre (JAX 008610). mTmG mice (JAX 007676) were bred with multiple Cre lines (40, 53). Floxed site primers were used to genotype as described below. Littermates and both sexes were used for all experiments, and mice were 8–12 weeks old at time of experiments. The use of littermate controls allowed for proper control of the microbiota as is standard in the field.

### scRNA-Seq analysis.

ScRNA-Seq mining was done using the Broad Institute’s Single Cell Portal database. All clusters remained the same as the original manuscript’s analysis (31). Human data were mined from the Gut Cell Atlas (https://www.gutcellatlas.helmsleytrust.org) (7).

### Colonic explants.

Explants were generated by removing the entire colon or the distal 15 cm of the SI. Any remaining connective tissue was removed from the outside of the tissue and then tissue was gently flayed open. Fecal and mucus contents were gently removed with forceps and placed in a 6-well plate with 1× PBS. After a brief wash with 1× PBS, tissues were placed in 15 mL Falcon tubes (VWR) in a tissue culture hood with 5 mL of complete RPMI 1640 medium (10% FBS, HEPES, sodium pyruvate, nonessential amino acids, 100 U/mL penicillin/streptomycin, and l-glutamate). Tubes were placed horizontally in a container and shaken for 25 minutes at room temperature (RT), at a speed sufficient to maintain gentle movement of the tissue. After this incubation, tissues were plated in 10 cm dishes with 20 mL of complete exosome-depleted RPMI (the same media as complete RPMI above but supplemented with 10% exosome-depleted FBS, Systems Biosciences) and then placed in a 37°C 5% CO_2_ incubator for 16 hours. The supernatant of these plates was used to isolate vesicles, as described below.

### NTA.

Size and concentration of isolated particles were determined using the Nanosight NS300 (Malvern Technologies). For each sample, three 60-second videos were taken at 25 frames per second. The detection threshold was maintained for each sample to ensure consistent measurement of particles.

### Vesicle isolation.

Vesicles were isolated as described (26). Briefly, supernatants were centrifuged for 10 minutes at 1,000*g*, then 10 minutes at 2,000*g*, both at 4°C (Eppendorf 5810 R centrifuge with A-4-81 rotor). Supernatants were transferred to Oak Ridge tubes (Thermo Fisher Scientific) and spun for 30 minutes at 10,000*g* and 70 minutes at 100,000*g*, both at 4°C, in a Thermo Fisher Scientific Sorvall Lynx 6000 with a T26-8 × 50 rotor. Pellets were washed with 25 mL of 1× PBS and spun at 100,000*g* for 70 minutes at 4°C. After removing PBS, pellets were resuspended in 0.02 μM filtered 1× PBS. To purify colonic EVs for miRNA sequencing, we resuspended the final pellet in 30% iodixanol gradient instead of PBS (65). A base solution of 60% iodixanol (in water, 0.5 M sucrose, 20 mM Tris pH 7.4, and 2 mM EDTA) was made followed by working solutions (WSs) of 40%, 30%, and 20%. The 30% solution was the basis of the gradient (2.5 mL), followed carefully by 1.05 mL of 20% (from 40% WS), and then finally 0.95 mL of 10% (from 20% WS). We spun this at 350,000*g* for 1 hour at 4°C with no brakes in a swinging bucket SW55Ti rotor (Beckman Optima L-90k Ultracentrifuge). We analyzed 500 μL fractions by NTA, and fraction 2 showed enrichment for vesicle-sized particles and was the expected density. This was then spun down at 100,000*g* for 30 minutes at 4°C with no brake, then resuspended in a small volume, and 700 μL QIAzol (QIAGEN) was added for further RNA analysis.

### mLN tissue prep.

mLNs were harvested and placed on a 40 μM filter (Thermo Fisher Scientific) in a 6-well plate with 5 mL complete RPMI. After being dissociated through the filter with the back end of a sterile 1 mL syringe, cells were spun down in 15 mL Falcon tubes at 400*g* for 5 minutes and then resuspended in 1 mL complete RPMI 1640 before cell counting with trypan blue solution (VWR). Similar cell numbers were plated for flow analysis.

### Colonic lamina preparation.

Immune cells were harvested as described (41, 66, 67). Briefly, murine colons with attached ceca were harvested and placed in 1× PBS in 6-well plates on ice. Remaining fat, connective tissue, and the cecum were removed. Colons were splayed and mucus and feces were removed from the colon before returning to the plate. Once all colons were cleaned, each colon was diced on a Petri dish lid using a razor blade and placed in a separate 50 mL tube. A total of 10 mL of prewarmed dissociation solution (1× HBSS without Ca^+^ and Mg^+^, 1.5 mM DTT, 10 mM HEPES, and 30 mM EDTA) was added and the tubes vortexed. Colons were incubated for approximately 25 minutes at 37°C with shaking at 150 rpm until solutions were cloudy from IECs’ separation. Samples were vigorously shaken 3 times, vortexed for 15 seconds, then poured over a 100 μM filter on a 50 mL conical tube to remove nonimmune cells such as IECs, and the filters were rinsed briefly with 2 mL of ice-cold 1× PBS. The remaining tissue was collected on the filter with forceps and moved to a new 50 mL conical tube. A total of 15 mL of prewarmed digestion solution (1× HBSS with Ca^+^ and Mg, 5% FBS, DNase, collagenase D, and dispase) was added. Samples were vortexed and incubated for 45 minutes at 37°C with shaking at 150 rpm until solution became cloudy. Samples were shaken 3 times and vortexed for 15 seconds. To collect the remaining immune cLP cells, the digested tissue was then poured over a 40 μM filter on a 50 mL conical tube containing 10 mL 1× PBS. Samples were next rinsed with 2 mL of ice-cold 1× PBS and spun at 800*g* for 10 minutes at 4°C. Next, supernatant was removed and discarded with vacuum. Cells were then resuspended in 500 μL of complete RPMI 1640 and counted following trypan blue exclusion staining.

### qPCR.

RNA was isolated following manufacturer protocols using the QIAGEN miRNeasy Mini or Micro Kit. Vesicle samples were prepped with Micro kits due to low RNA concentration. Mouse tissue and cell isolates were prepped with Mini kits due to a higher abundance of RNA. cDNA was generated according to manufacturer’s protocols with qScript cDNA SuperMix (QuantaBio) for mRNA (using 400 ng RNA input) and miRNA LNA RT kit (QIAGEN) for miRNA reactions (100 ng RNA input). All miR primers were predesigned and ordered from QIAGEN. qPCR reactions used PowerUp SYBR Green Master Mix (Applied Biosciences) according to manufacturer’s protocol and 500 nM primer reaction mixes generated from primer stocks synthesized at the University of Utah DNA/Peptide Facility (Table 2). All reactions were run on an Applied Biosciences QS6 Thermocycler.

### DSS colitis.

Animals were given 2.5% *w/v* DSS (MP Biomedicals MW 36,000–50,000) in their drinking water, replaced every other day. Mouse weight was measured daily and if animals reached 80% of their starting weight, they were culled. Colon length was measured from rectum to below the cecum.

### αIL-10R colitis.

Mice received i.p. injection of 1 mg mAb/100 μL/20 g mouse weight (Bio X Cell; catalog BE0050) (34). Mice were monitored for weight loss and rectal prolapse over 5 weeks, biweekly. Mice were culled if body weight was 20% less than starting weight.

### BM chimera.

Mice were lethally irradiated as described previously (27). After irradiation, mice were retro-orbitally injected with 10 million RBC-depleted BM cells isolated as below. Mice reconstituted their hematopoietic systems for 12 weeks before isolating CD11b^+^ and CD3^+^ cells by FACS from the cLP and mLNs.

### Serum isolation.

Serum was isolated from mouse heart blood kept on ice until centrifugation. Spins at 500*g* were done twice for 10 minutes each and supernatants saved at each time. Serum was stored at –80°C until used for ELISAs.

### Colon histology and scoring.

Colons were fixed as done previously (66, 67) within histology cassettes overnight at RT in 10% buffered formalin phosphate solution after removal of fecal contents and then transferred to 70% ethanol at 4°C until staining. Sectioning and H&E staining were done at the Huntsman Cancer Institute (HCI) Biorepository and Molecular Pathology Shared Resource in the ARUP operated Research Histology division. DSS colon scoring was done in a blinded manner with the following rubric scales: percentage of colon crypt loss (0.5 [5%] to 7 [>75%]), crypt loss severity (1 [partial loss] to 5 [full loss]), and inflammatory aggregates (1 [1 to 3] to 3 [>7]). For αIL-10R colon scoring, colons were prepared in the same manner as DSS colitis. Additionally, colons were stained for Alcian blue to assess goblet cell loss (34, 36). Scoring was done in a blinded fashion with the following scale: epithelial hyperplasia — 0 (none), 1 (minimal), 2 (mild), 3 (moderate), 4 (severe); goblet cell loss — 0 (none), 1 (slight depletion), 2 (mild), 3 (moderate), 4 (few mucin-containing cells remaining); inflammatory aggregates — 0 (none), 1 (1–2 in LP), 2 (several mononuclear cell infiltrates in LP), 3 (involves submucosa, rarely transmural), 4 (transmural); and crypt abscesses — 0 (none), 1 (small epithelial cell erosions), 2 (ulcers observed), 3 (crypt abscesses and ulcers present). QuPath software was utilized to measure epithelial cell crypt depth with measurements taken along the entire colon length; additionally, inflammatory aggregates were quantified.

### Flow cytometry.

Samples were blocked with CD16/32 Abs (BioLegend catalog 101302) for 20 minutes at 4°C. After pelleting at 400*g* for 5 minutes at room temperature, cells were stained for 30 minutes in the dark at 4°C. Dilutions of BioLegend antibodies (unless otherwise noted) are as follows: splenocyte FACS (Figure 1): CD3-PacBlue (100334; 1:250), B220-FITC (103206, 1:500), CD11b-PerCP/Cy5.5 (101228, 1:1,000), CD4-PE (100408, 1:2,000), and CD8-APC (100712, 1:250). Macrophage panel (Figures 2, 3, and 6): CD45-FITC (103108), CD64-BV711 (139311), CD11c-PerCP/Cy5.5 (117328), CD11b-BV605 (101237), MHCII-PacBlue (107620), CD38-PE/Cy7 (102717) (all 1:250), and Egr2-APC (eBioscience 17-6691-82, 1:50). Th17 panel (Figures 2, 3, and 5): CD45-FITC, CD3-APC/Cy7 (100222), CD4-PacBlue, IL-17-APC (eBioscience 17-7177-81), and RORγT-PE 610 (eBioscience 61-6981-82) (all intracellular stains 1:50, all surface stains 1:250). ImageStream (Figure 5): CD45-PE/Cy7 (103114), CD3-APC/Cy7, CD4-Pac Blue, CD11b-BV605, and Ghost Dye – BV510 (Tonbo Biosciences 13-0870-T100). After staining, cells were washed twice for 5 minutes each with column buffer (1× HBSS without Ca and Mg, HEPES, EDTA, and FBS). Surface stain–only cells were fixed overnight with 1% paraformaldehyde and washed in column buffer twice before analysis. If intracellular staining was necessary, the FoxP3/Transcription Factor Staining Buffer Kit (Tonbo Biosciences) and protocol was followed. All samples were then resuspended in 300 μL of column buffer and run on a BD LSR Fortessa after compensating with UltraComp eBeads (Thermo Fisher Scientific) and using single-stain controls.

### ELISA.

Lipocalin-2 ELISAs were performed according to manufacturer’s protocol (Thermo Fisher Scientific DY1857-05). Dilutions of serum were made according to sickness of animals but ranged from 1:500 to 1:1,000.

### miRNA-sequencing/bioinformatic analysis.

miRNA-sequencing and bioinformatic analyses were done as described previously using the University of Utah Bioinformatic Analysis core services (68). The cutoffs for miRNAs used for comparison between colon and DCs were *P* adj. < 0.05 and base level detection of at least 100.

### ImageStream analysis.

mLN samples were run on the Amnis ImageStream MKII Imaging Flow Cytometer using 405, 488, 561, 642, and 785 nm excitation lasers and 60× original magnification. Analysis was done with IDEAS software, and cells were gated based on their bright-field gradient RMS (average slope spanning 3 pixels in an image) as a measure of focus, followed by their area and aspect ratio to exclude debris and aggregates. CD45^+^CD3^+^CD4^+^ or CD45^+^CD3^–^CD11b^+^ cells were analyzed for tdTomato surface staining and GFP^+^ puncta.

### FACS.

Isolated vesicles from ultracentrifugation were diluted in 1 mL of 0.02 M filtered 1× PBS and sorted on a BD FACSAria cell sorter using the violet laser as the side scatter (SSC) and setting the forward scatter and SSC on a logarithmic scale. Fluorescent beads of known sizes (110 nm and 500 nm, SBI EXOF300A-1) were used to ensure properly sized vesicles were isolated. After sorting based on membrane fluorescence, vesicles were spun down at 100,000*g* in 5 mL of 1× PBS (0.02 M filtered) for 30 minutes, and the pellet was resuspended in QIAzol for RNA isolation and miRNA qPCR.

### BMDCs/BMDMs and exosome administration to mice.

BMDCs and BMDMs were generated as previously described (27, 69). Briefly, 10 million BM cells were plated in 15 cm dishes with 20 mL of complete RPMI supplemented with GM-CSF at 20 ng/mL. After 4 days, new GM-CSF was added in 10 mL of complete RPMI. After 7 days, vesicles were isolated with ultracentrifugation. For injection experiments, 10^9^ vesicles were injected i.p. after size and concentration confirmation by NTA on days 2, 4, and 6 after start of DSS treatment. Vesicles were sex-matched, when possible; otherwise only female BMDC-derived vesicles were used to prevent potential immune stimulation by Y antigen. For BMDMs, a total of 6 million BM cells were plated in 10 cm dishes with 10 mL of complete DMEM (penicillin/streptomycin, l-glutamine, FBS) supplemented with M-CSF at 20 ng/mL. After 4 days, new M-CSF was added in an additional 5 mL of complete DMEM. All cells were generated from mouse lines described in this study.

### Human samples.

All specimens were of colonic origin and diagnoses were confirmed by clinical criteria. Non-IBD (control) tissues were obtained from histologically normal, noninflamed large bowel specimens from patients admitted for bowel resection because of conditions including colon cancer, benign polyps, diverticulitis, and colonic inertia. The mucosal layer of each colon was dissected and washed, and surface debris was removed by blotting the tissue with paper towels multiple times. The mucosal layer was then removed and cut into small strips (~0.5 cm) and snap-frozen in liquid nitrogen. All specimens were age and sex matched.

### Schematics.

All schematics and graphical abstract were created with BioRender.com.

### Statistics.

All statistical analysis and graphing were done in GraphPad Prism 9.0 software. Data represent mean ± SEM. *P* > 0.05 was nonsignificant, *P < 0.05, **P < 0.005, ***P < 0.0005.

### Study approval.

Human intestinal tissue specimens were obtained within 2 hours after resection from patients undergoing surgery from the Department of Surgical Pathology at the Cleveland Clinic under an IRB-approved protocol. Mice were housed in a vivarium with a 12-hour light/12-hour dark cycle per day, temperatures of 22°C, and 20% relative humidity. Experimental mouse procedures and husbandry were performed with the approval of the IACUC of University of Utah.

Additional methods details are in Supplemental Methods.

## Author contributions

Experiments were designed by KMB, DMW, ACP, WPV, WZS, JLR, and RMO and completed by KMB, MCN, WWT, TRC, DGB, AG, SHL, AMW, JHH, JKM, JEM, KAK, AGR, VBT, and JWT. Data analysis was done by KMB, MCN, WWT, TRC, DGB, and AG. Human samples were provided by ACP. Writing of the manuscript was done by KMB, JLR, and RMO and editing by KMB, MCN, DGB, DMW, WPV, WZS, MA, JLR, and RMO. This study was supervised by JLR and RMO.

## Supplementary Material

Supplemental data

## Figures and Tables

**Table 1:**
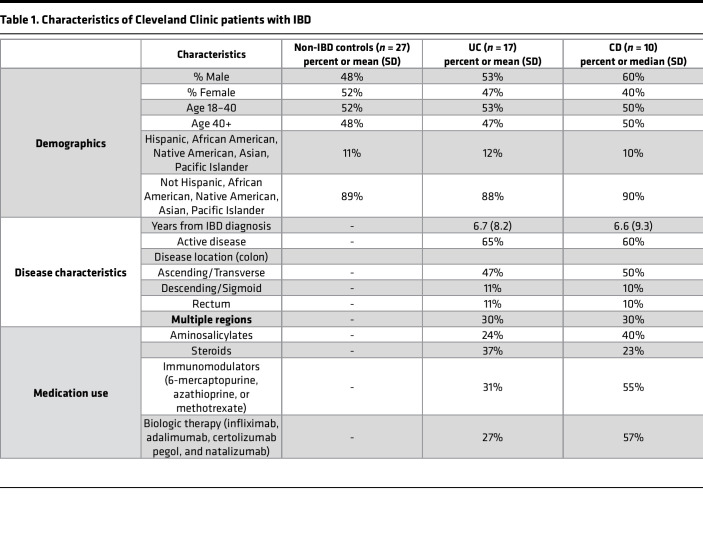
Characteristics of Cleveland Clinic patients with IBD

**Table 2:**
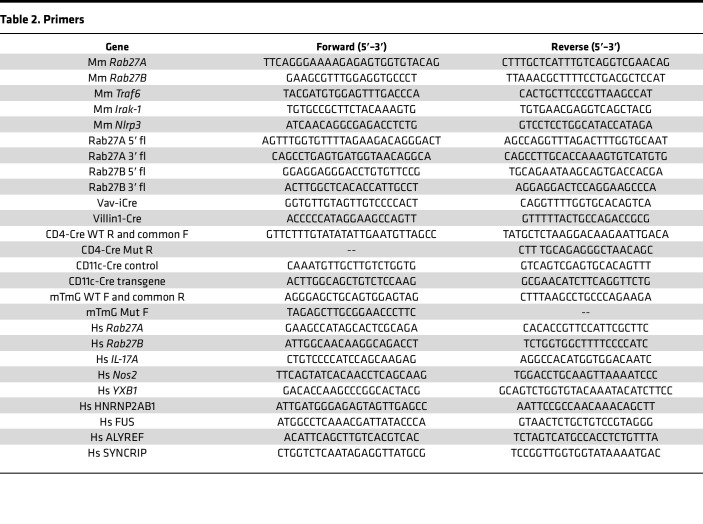
Primers

## Version 1 10/10/2022

Electronic publication
