# Supplementary material for: CD11c+ myeloid cell exosomes reduce intestinal inflammation during colitis
Source: JCI Insight. 2022 Oct 10;7(19):e159469. doi: 10.1172/jci.insight.159469 (PMC9675566; doi:10.1172/jci.insight.159469)
Supplement: Supplemental data [file jciinsight-7-159469-s043.pdf]

Supplementary Materials

Methods

Fig. S1. Generation of novel Rab27A and Rab27B floxed mouse animals

Fig. S2. Baseline Vav-Rab27A cKO immune cell populations and confirmation of various cKOs

Fig. S3. Baseline CD11c-Rab27A cKO immune cell populations and confirmation of cKOs

Fig. S4. In vivo transfer of miR-146a by BMDC EVs regulates macrophage skewing

Fig. S5. Human CD patients have dysregulated transcription of key exosome loading machinery, cargo and secretion proteins

Supplemental Materials

Methods

Genotyping:

DNA was isolated from mouse ear punches using the ThermoFisher Red Extract System. The following PCR programs were run for indicated genes: *Rab27A fl*: 94°C for 5:00; 30 cycles of 94°C 0:30, 62°C for 0:30, 72°C for 1:00; 72°C 10:00, 4°C hold. *Rab27B fl*: 94°C for 5:00; 35 cycles of 94°C 0:30, 62°C for 0:30, 72°C for 1:00; 72°C 10:00, 4°C hold. *Villin1-Cre*: 95°C for 2:00; 40 cycles of 95°C 0:20, 58°C for 0:20, 72°C for 0:45; 72°C 2:00, 4°C hold. *CD4-Cre*: 95°C for 5:00; 30 cycles of 95°C 1:00, 56°C for 1:30, 72°C for 2:00; 72°C 8:00, 4°C hold. *Vav-iCre:* 94°C for 5:00; 40 cycles of 94°C 0:20, 64°C for 0:20, 72°C for 0:45; 72°C for 5:00, 4°C hold. *CD11c-Cre*: 95°C, 5:00; 10 cycles of 94°C 20 sec, 60°C for 0:15 (-0.5°C / cycle), 68°C for 0:10; 94°C 0:15, 55°C 0:15, 72°C 0:10; 72°C 2:00, 4°C hold. *mTmG fl*: 95°C, 5:00; 10 cycles of 94°C 0:20, 65°C for 0:15 (-0.5°C / cycle), 68°C for 0:10; 94°C 0:15, 60°C 0:15, 72°C 0:10; 72°C 2:00, 4°C hold. PCR products were visualized using 2% gel electrophoresis.

Fecal LCN2

For fecal LCN2 isolation, feces were weighed in a pre-weighed eppie tube. For every 10 mg of feces, 10 ul of 1X HBSS with Ca, Mg was added. The fecal pellet was then mashed with a 1000 ul pipette tip. This was vortexed and spun down at 60 x g for 5 minutes. The supernatant was transferred to a new tube and spun at 8000 x g for 5 minutes and supernatant containing free LCN2 was saved.

Immunoblotting

Cellular pellets were resuspended in 100 µl of fresh protein lysis buffer (1X RIPA and 1% protease inhibitor) and sonicated with the following program: 20 seconds on, 30 seconds off, for 5 cycles. Tubes were spun at 21,000 x g for 3 minutes at 4C and supernatant was retained. Protein concentration was measured (Pierce BCA Protein Assay Kit). Sonicated proteins were separated via SDS-PAGE and transferred to a 0.45 µM nitrocellulose membrane O/N at 4 C. After blocking in 1% BSA, antibody staining was performed with CD9 (Abcam EPR2949), CD63 (Santa Cruz Biotechnology, sc-15363), Rab27A (Cell Signaling Technology, D7Z9Q), Rab27B (Proteintech 13412-1-AP) and mouse β-Actin (Cell Signaling Technology, 3700S) antibodies. An HRP conjugated secondary antibody was detected with Amersham ECL reagent (GE) with a BioRad GelDoc and ImageLab 6.1 Software.

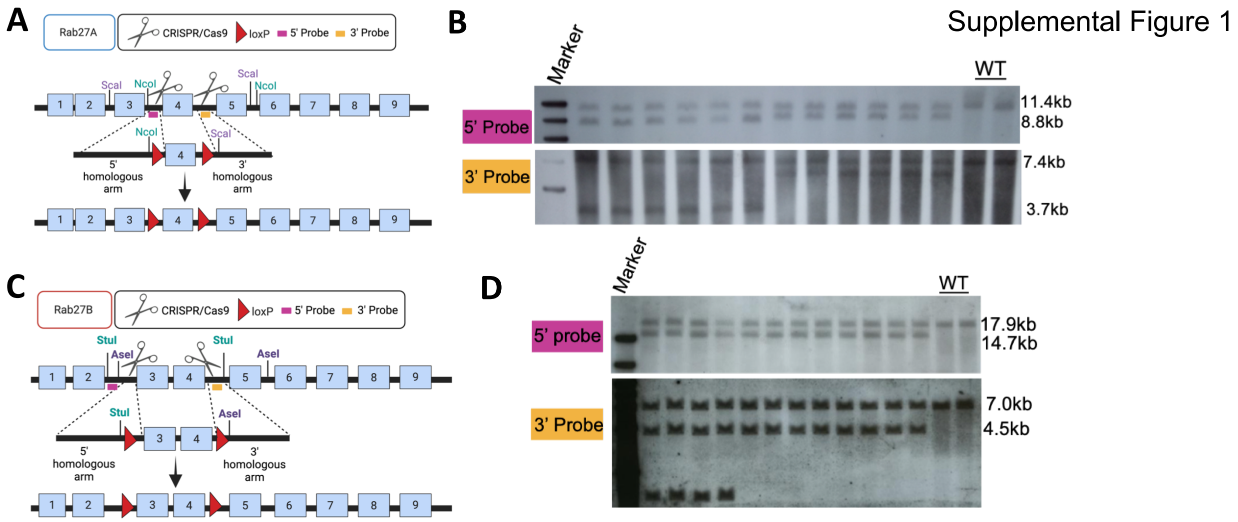

Supplemental Figure 1: Generation of novel Rab27A and Rab27B floxed mouse animals
A) Schematic of loxP site insertion for *Rab27A* by CRISPR/Cas9 technology. **B)** Southern blot for 5' and 3' probe to select founder mice of floxed lines. **C)** Schematic of loxP site insertion for Rab27B. **D)** Southern blot for 5' and 3' probe to select founder mice of floxed lines.

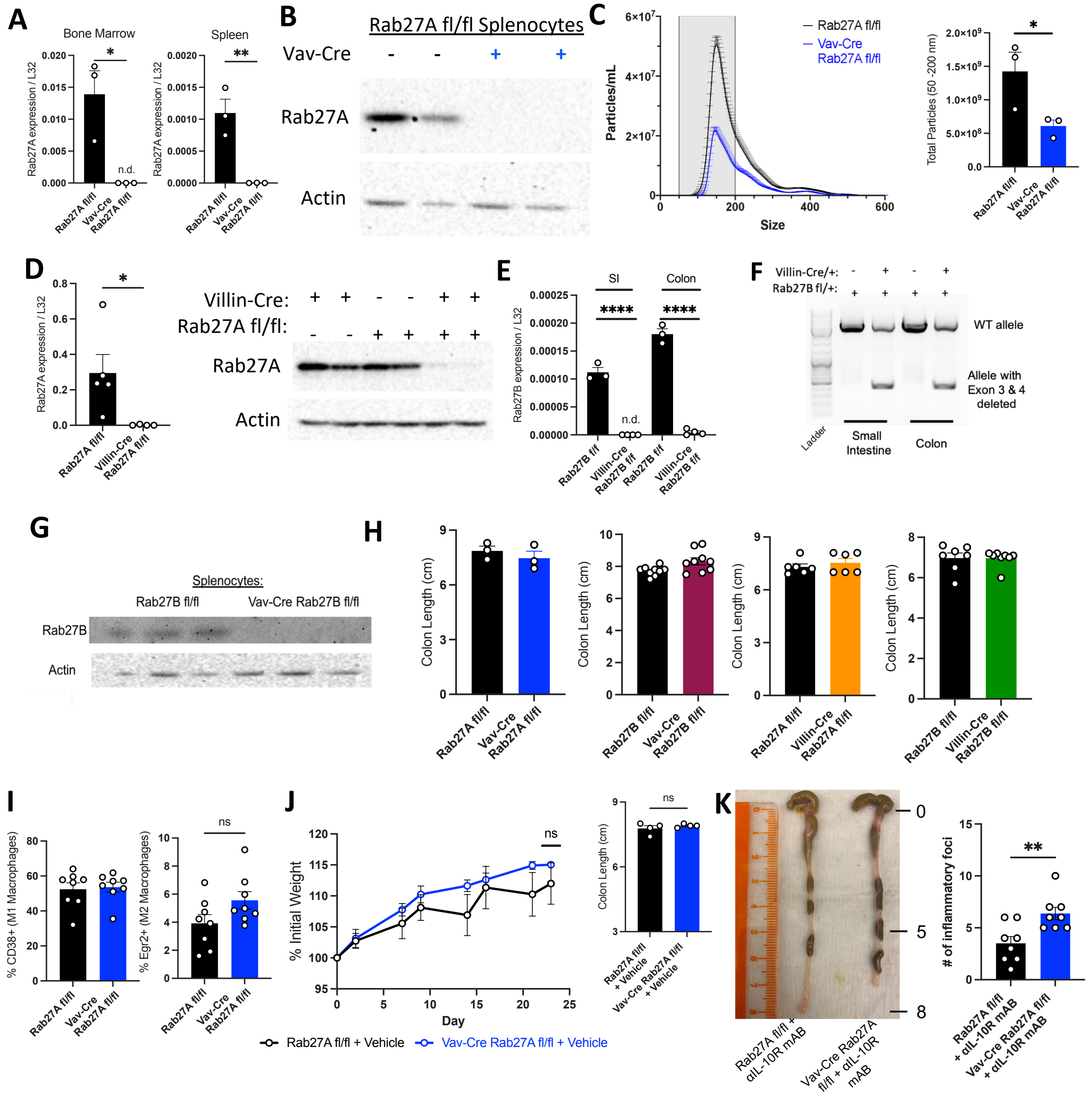

Supplemental Figure 2: Baseline Vav-Rab27A cKO immune cell populations and confirmation of various cKOs

A) *Rab27A* expression in BM cells and splenocytes of WT and KO animals (n=3). **B)** IB of Rab27A and Actin within baseline splenocytes of WT and Vav-iCre Rab27A fl/fl mice. **C)** NTA of vesicles isolated from WT and Vav-Rab27A cKO BMDCs. Exosome sized (50-200 nm) particle quantification from NTA (n=3)**. D)** *Rab27A* expression in IECs from floxed and Villin-Rab27A cKO mice and IB for Rab27A and Actin in Villin-Cre only, floxed and Villin-Rab27A cKO IECs (n=4). **E)** *Rab27B* expression in IECs of the SI and colon of WT and Villin-Rab27B

cKO mice (n=3). **F)** PCR analysis of new floxed allele with exons 3 & 4 deleted in the presence of Villin-Cre in SI and colon IECs. **G)** IB of Rab27B and Actin in floxed and Vav-Rab27B cKO splenocytes (n=3). **H)** Baseline colon lengths of all cKO lines in Figure 2. **I)** % of M1 and M2-like macrophages in the cLP of baseline WT and Vav-Rab27A cKO animals. **J)** Weight and colon length of control mice from Figure 2Q given only vehicle. **K)** Representative colon images and length from Figure 2Q and quantification of inflammatory aggregates. Unpaired two-tailed t test for all bar graphs.

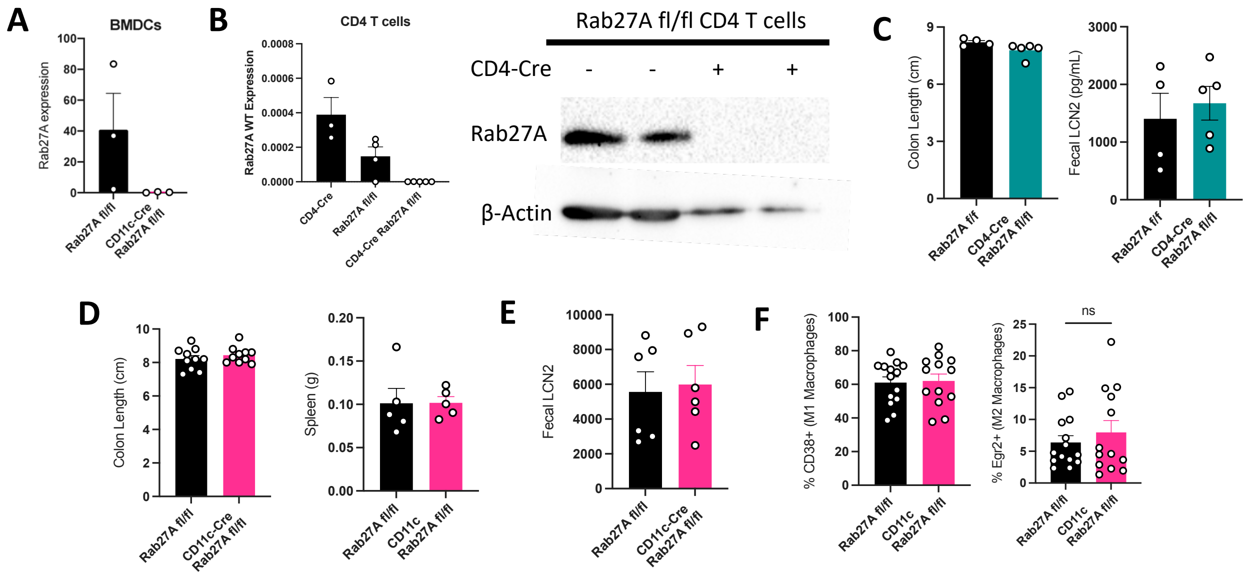

Supplemental Figure 3: **Baseline CD11c-Rab27A cKO immune cell populations and confirmation of cKOs**

A) *Rab27A* expression in BMDCs from WT and CD11c-Rab27A cKO mice. **B)** *Rab27A* expression in column sorted CD4 T cells from CD4-Cre, floxed and CD4-Cre Rab27A fl/fl animals (n= 3-5). IB of Rab27A and Actin in sorted CD4+ T cells from floxed or CD4-Rab27A cKO animals. **C)** Baseline colon length and fecal LCN2 levels from WT and CD4-Rab27A cKO. **D)** Baseline colon length and spleen weight of WT and CD11c-Rab27A cKO mice. **E)** Baseline fecal LCN2 of WT and CD11c-Rab27A cKO animals. **F)** Baseline percentage of CD38+ M1-like macrophages and Egr2+ M2-like macrophages in floxed and CD11c-Rab27A cKO mLNs.

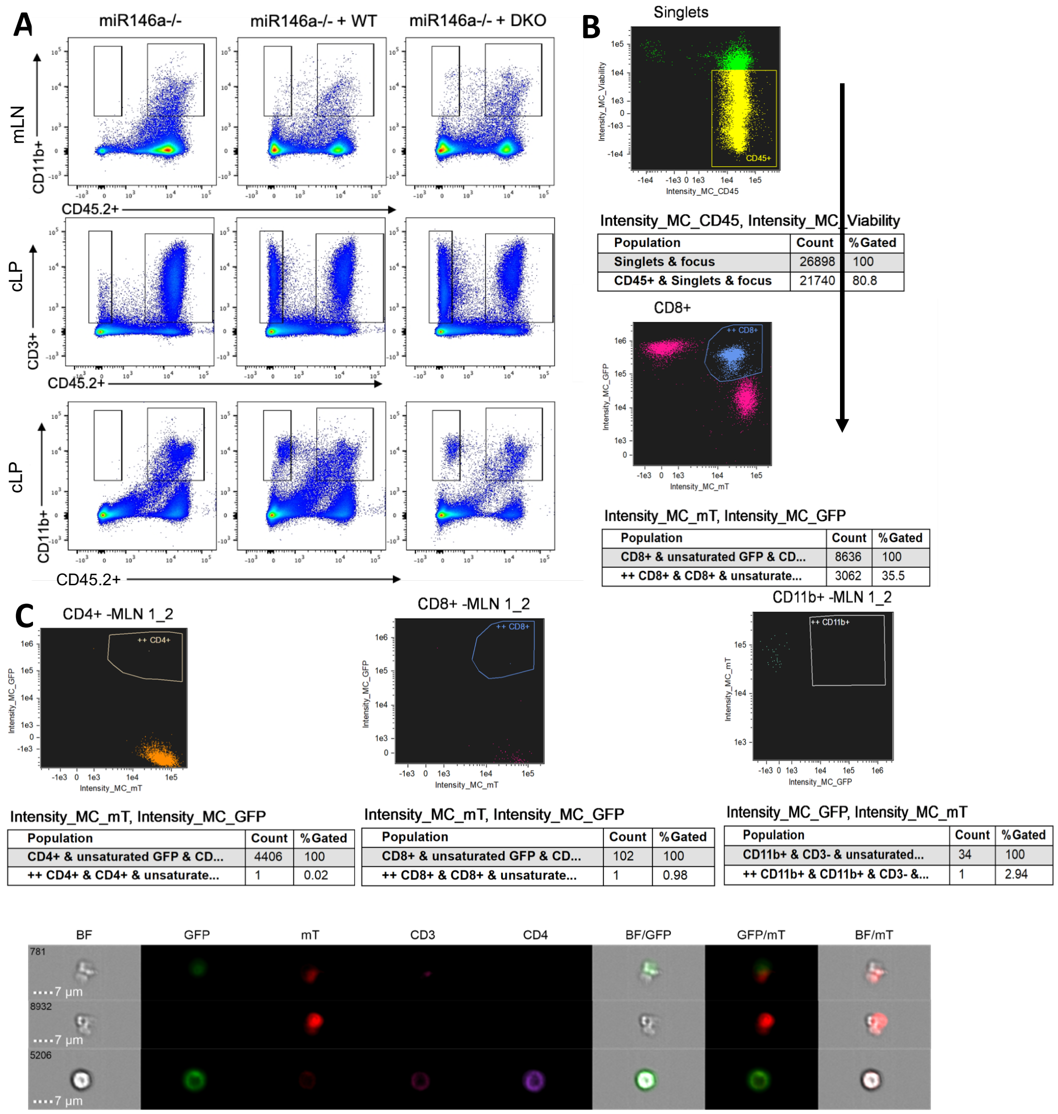

Supplemental Figure 4: In vivo administration of miR-146a by BMDC EVs regulates macrophage skewing

A) Representative flow plots of sorted CD45.2+ cells from intestinal tissues. **B)** Gating strategy for Imagestream analysis, progressing from Singlets, selecting CD45+, Ghost Dye- and then gating on CD3+ CD4 or CD8+ and then mTomato+ and mGFP+. **C)** Representative plots of mTmG fl/fl animals as negative controls, any doublets are shown.

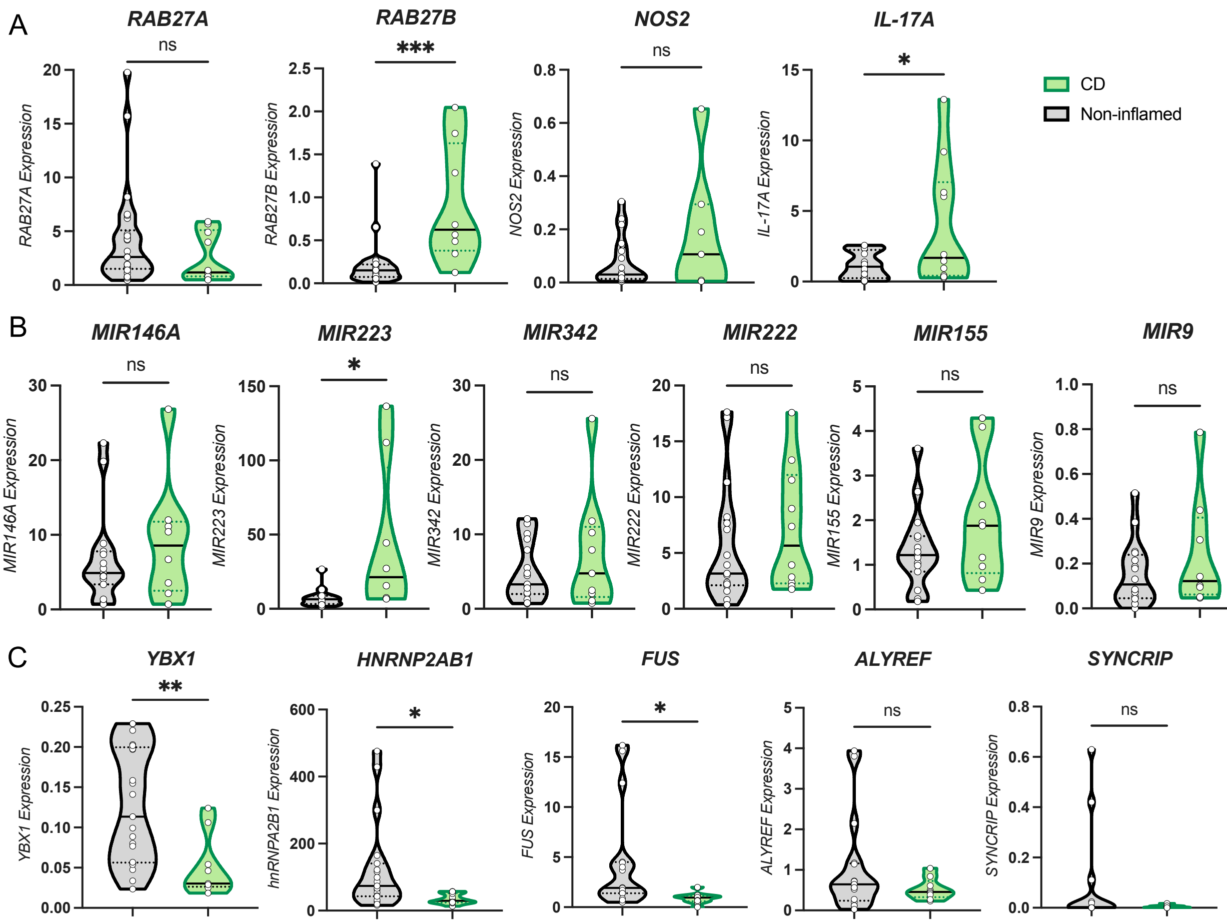

Supplemental Figure 5: CD patients have dysregulated expression of key exosome loading machinery, cargo and secretion proteins

A) qPCR of *RAB27A*, *RAB27B*, *IL-17A* and *NOS2* in non-IBD (n = 27) and CD patient (n = 10) colonic samples. **B)** miRNA qPCR of miR-146a, miR223, miR-342, miR-222, miR-155 and miR-9 in **A.** **C)** qPCR of *YBX1, HNRNP2AB1*, *FUS, ALYREF* and *SYNCRIP* in **A**. Unpaired two-tailed t test of all violin plots.
